# Assessing the practical differences between model selection methods in inferences about choice response time tasks

**DOI:** 10.3758/s13423-018-01563-9

**Published:** 2019-02-19

**Authors:** Nathan J. Evans

**Affiliations:** Department of Psychology, University of Amsterdam, Amsterdam, The Netherlands

**Keywords:** Model selection, Decision-making, Response time modeling, Bayes factors, Predictive accuracy

## Abstract

Evidence accumulations models (EAMs) have become the dominant modeling framework within rapid decision-making, using choice response time distributions to make inferences about the underlying decision process. These models are often applied to empirical data as “measurement tools”, with different theoretical accounts being contrasted within the framework of the model. Some method is then needed to decide between these competing theoretical accounts, as only assessing the models on their ability to fit trends in the empirical data ignores model flexibility, and therefore, creates a bias towards more flexible models. However, there is no objectively optimal method to select between models, with methods varying in both their computational tractability and theoretical basis. I provide a systematic comparison between nine different model selection methods using a popular EAM—the linear ballistic accumulator (LBA; Brown & Heathcote, Cognitive Psychology *57*(3), 153–178 2008)—in a large-scale simulation study and the empirical data of Dutilh et al. (Psychonomic Bulletin and Review, 1–19 2018). I find that the “predictive accuracy” class of methods (i.e., the Akaike Information Criterion [AIC], the Deviance Information Criterion [DIC], and the Widely Applicable Information Criterion [WAIC]) make different inferences to the “Bayes factor” class of methods (i.e., the Bayesian Information Criterion [BIC], and Bayes factors) in many, but not all, instances, and that the simpler methods (i.e., AIC and BIC) make inferences that are highly consistent with their more complex counterparts. These findings suggest that researchers should be able to use simpler “parameter counting” methods when applying the LBA and be confident in their inferences, but that researchers need to carefully consider and justify the general class of model selection method that they use, as different classes of methods often result in different inferences.

**Electronic supplementary material:**

The online version of this article (10.3758/s13423-018-01563-9) contains supplementary material, which is available to authorized users.

## Introduction

Over the past several decades, formalized cognitive models have become a dominant way of expressing theoretical explanations and analyzing empirical data. These formalized models take the verbal explanations of theories of cognitive processes, and express them as precise mathematical functions that make exact quantitative predictions about empirical data. Formalized cognitive models have been largely adopted within the area of rapid decision-making, with the evidence accumulation models (EAM) forming the most dominant class of models (see Ratcliff, Smith, Brown, & McKoon, 2016 for a review). EAMs propose that decisions are the result of evidence from the stimuli accumulating over time for each potential decision alternative (at a rate called the “drift rate”), until the evidence for one of the alternatives reaches a threshold level of evidence (called the “decision threshold”) and a response is triggered (Stone, 1960; Ratcliff, 1978; Usher & McClelland, 2001; Brown & Heathcote, 2008).

One of the primary uses of EAMs has been as a “measurement tool” for empirical data analysis, in order to provide more nuanced answers to research questions in terms of the decision-making process, rather than in terms of observed variables (Donkin, Averell, Brown, & Heathcote, 2009). These applications have been widespread, and have greatly improved our understanding of psychological theory in many instances. For example, a common finding in the aging literature has been that older adults are slower than younger adults in many cognitive tasks, with an initial—and prominent—explanation of this phenomenon being the “cognitive slowdown” account, where older adults had slower mental processing than younger adults (Salthouse, 1996). Using EAMs, Ratcliff, Thapar, and McKoon (2001) compared this account (i.e., older adults have lower drift rates than younger adults) to an alternate explanation: that older adults are simply more cautious (i.e., have higher thresholds) than younger adults. Interestingly, Ratcliff et al.,’s findings indicated that in many tasks, older and younger adults have near identical drift rates, though older adults are much more cautious, providing strong evidence against the general “cognitive slowdown” account. EAMs have been used to answer a wide variety of similar research questions across a large number of fields and areas, including intelligence (van Ravenzwaaij, Brown, & Wagenmakers, 2011), alcohol consumption (van Ravenzwaaij, Dutilh, & Wagenmakers, 2012), discrete choice decision-making (Hawkins et al., 2014b), stop-signal paradigms (Matzke, Dolan, Logan, Brown, & Wagenmakers, 2013), absolute identification (Brown, Marley, Donkin, & Heathcote, 2008), optimality studies (Starns & Ratcliff, 2012; Evans & Brown, 2017; Evans, Bennett, & Brown, 2018), personality traits (Evans, Rae, Bushmakin, Rubin, & Brown, 2017), and a range of neuroscience data (Forstmann et al., 2008, 2011; Turner, Forstmann, et al., 2013).

In order to use EAMs to decide between different theoretical accounts, such as the “cognitive slowdown” and “increased caution” accounts of slower task performance for older adults discussed above, a method is needed to decide which account is best supported by the empirical data. However, this is not necessarily the account that most closely matches the data (i.e., the “goodness-of-fit”), as more flexible models can predict a wider range of potential data patterns, and are able to “over-fit” to the noise in the sample data, which can often result in a more flexible model more closely matching the data than a simpler, but more correct, model (Myung & Pitt, 1997; Myung, 2000; Roberts & Pashler, 2000; Evans, Howard, Heathcote, & Brown, 2017). Therefore, selecting between models requires a careful balance between goodness-of-fit and flexibility, a process commonly referred to as “model selection”, which is commonly performed through some quantification of these two criteria, and expressed as some metric.

Unfortunately, there is no objectively optimal way to select between models, with methods varying in both their computational tractability and theoretical basis. Therefore, the inferences that researchers draw about psychological theory, such as the aging example above, can depend on their choice of method to select between the competing models. The simplest, most computationally tractable metrics commonly used to perform model selection are the Akaike Information Criterion (AIC; Akaike, 1974) and the Bayesian Information Criterion (BIC; Schwarz, 1978), with the *χ*^2^ test of relative deviance also being a well-known method for the case of nested models. These metrics only require simple frequentist methods of parameter estimation, using the maximum likelihood estimate as the measure of goodness-of-fit, and a transformation of the number of free parameters in the model as a measure of the flexibility (commonly referred to as “parameter counting”). However, parameter counting ignores “functional form flexibility”: how the model’s specific function alters the amount of flexibility provided by each parameter, and therefore, the model overall (Myung, 2000; Evans et al., 2017). This is particularly relevant for models that contain highly correlated parameters, such as EAMs, as the correlation in the parameters often reduces the overall flexibility of the model. These limitations have resulted in researchers adopting more sophisticated, computationally taxing methods of model selection, such as the Deviance Information Criterion (DIC; Spiegelhalter, Best, Carlin, & Van Der Linde, 2002) and the Widely Applicable Information Criterion (WAIC; Vehtari, Gelman, & Gabry, 2017). These metrics both require Bayesian parameter estimation, with goodness-of-fit being calculated as the average likelihood of the data given the parameters (i.e., *p*(*y*|***𝜃***)) over the entire posterior, and flexibility being calculated by some measure of variability of the likelihood over the posterior. In addition, recent research has highlighted methods that can provide accurate approximations of the Bayes factor in complex cognitive models within a reasonable time-frame (Evans & Brown, 2018; Gronau et al., 2017; Annis, Evans, Miller, & Palmeri, 2018; Evans & Annis, 2019), with the Bayes factor commonly argued to provide the optimal balance between goodness-of-fit and flexibility within model selection. Bayes factors involve integrating the unnormalized posterior probability (i.e., *p*(*y*|***𝜃***)*p*(***𝜃***)) over the entire parameter space, rewarding models for having a high posterior probability, but penalizing models for having an overly wide set of a priori predictions (Kass & Raftery, 1995).

In addition to varying in computational tractability, these methods vary on their theoretical basis for selecting between models. AIC, DIC, and WAIC all attempt to find the model with the best “predictive accuracy”, which is the model that is able to best predict future empirical data, given some fixed set of parameter values or distributions (Akaike, 1974; Spiegelhalter et al., 2002; Vehtari et al., 2017). These methods punish models for over-fitting to the noise in sample data, as over-fitting will result in poor prediction of future data (Myung, 2000; Yarkoni & Westfall, 2017). In contrast, BIC and the Bayes factor attempt to find the model that provides the best explanation for the current data (though this can also be posed in terms of a different type of “predictive accuracy” [Bayesian updating], see Rouder & Morey, 2018), integrating over all possible parameter value—rather than the fixed set of parameter values/distributions in the predictive accuracy methods—with this integration punishing models for being able to predict large ranges of potential data trends (Schwarz, 1978; Kass & Raftery, 1995; Evans & Brown, 2018). Therefore, the choice of model selection method also has important underlying theoretical implications.

Alternatively, some researchers have argued that parameter estimation should be used to decide between theoretical accounts, rather than model selection metrics that attempt to balance goodness-of-fit and flexibility (Kruschke & Liddell, 2018). One of the most well-defined parameter estimation approaches to date is the Region Of Practical Equivalence (ROPE; Kruschke, 2011; Kruschke & Liddell, 2018), where a region that is viewed as being “practically zero” is defined a priori (i.e., the ROPE). The posterior distributions of the parameters are then estimated from the data (i.e., Bayesian parameter estimation), and if the 95% credible interval falls completely within the ROPE then the parameter is viewed as being zero. Alternatively, if the 95% credible interval falls completely outside of the ROPE, then the parameter is viewed as not being zero. However, any other result (e.g., partial overlap with the ROPE) is viewed as being “indeterminate”, meaning that the standard ROPE approach will only decide in favor of a theoretical account when certainty is high, and in other cases provide little information.

My study aims to provide the first systematic comparison between a range of model selection methods in their inferences between different theoretical accounts within EAMs, as a follow-up to the recent many-lab study of Dutilh et al., (2018). The study of Dutilh et al., (2018) attempted to assess the quality of inferences about response time data from expert researchers, where 17 teams of researchers were each able to pick a general approach of their choice for making assessments about empirical response time data, and these inferences were assessed for both consistency with the “selective influence” assumption (see Rae, Heathcote, Donkin, Averell, & Brown, 2014 for a more detailed discussion of this assumption), and consistency with one another. However, due to the vast range of approaches chosen by the relatively small number of teams (e.g., some teams opted for model-free, heuristic approaches), the inferences of Dutilh et al., (2018) were quite general, and specifics such as the impact of model selection method were not discussed. The current study aims to take a more systematic approach, assessing how these methods practically compare in realistic situations. Specifically, I use the Linear Ballistic Accumulator (LBA; Brown and Heathcote, 2008), a widely applied and computationally tractable EAM, to assess the inferences of nine common methods of model selection in both simulated and empirical data. Importantly, if there are few practical differences between the methods, then concerns surrounding functional form flexibility or the theoretical underpinnings of the methods are of little importance, and researchers should use the most computationally simple methods for greater efficiency when applying the LBA. However, if there are large differences, then researchers need to carefully consider the theory and level of sophistication behind different methods before choosing which one to use; a practice that does not seem to currently be commonplace.

## Method

### The linear ballistic accumulator

Due to the large-scale nature of my simulation study (2500 data sets, resulting in 10,000 total models being fit and 90,000 model selection metrics being calculated), I focused my assessment on a single, widely applied EAM: the linear ballistic accumulator (LBA; Brown & Heathcote, 2008; see Rae et al., 2014; Ho et al., 2014; Evans et al., 2017; Tillman, Benders, Brown, & van Ravenzwaaij, 2017; Evans, Steyvers, & Brown, 2018 for applications). The LBA is arguably the simplest EAM that provides inferences based on the entire choice response time distributions (Brown & Heathcote, 2008; though see Wagenmakers, Van Der Maas, & Grasman, 2007 and Grasman, Wagenmakers, & Van Der Maas, 2009 for simpler EAMs based on summary statistics), allowing the simulation study to be performed within a feasible computational time-frame.

The LBA proposes that decisions are based on the noiseless, leakless, independent accumulation of evidence for each alternative. The rate of evidence accumulation for each alternative (i.e., the drift rate) differs randomly between trials according to a truncated normal distribution (lower bound of 0), and the amount of starting evidence for each alternative differs randomly between trials according to a uniform distribution (lower bound of 0). In addition, some amount of time is assumed to be dedicated to non-decision related processes, such as perception and motor function (called “non-decision time”). The LBA contains five parameters per accumulator (i.e., for each alternative): the mean drift rate over trials (*v*), the standard deviation in drift rate over trials (*s*), the decision threshold (*b*), the upper bound of the uniform starting point distribution (*A*), and the non-decision time (*t*_0_). As is commonly the case in applications, I constrained the *t*_0_, *A*, and *b* parameters to be the same across accumulators, with each accumulator having a different mean drift rate (*v*_*c*_ and *v*_*e*_ for the accumulators reflecting the correct and incorrect responses, respectively) and standard deviation in drift rate (*sv*_*c*_ and *sv*_*e*_). Per convention, *sv*_*c*_ was fixed to 1 to satisfy a scaling property within the model (Donkin, Brown, & Heathcote, 2009).

To create different theoretical accounts within the LBA to compare in each data set, I defined four sub-models that allowed for different changes in parameter values across “within-subjects” experimental conditions. These were (1) a “null” model where all parameters were constrained to have the same values across conditions, (2) a “drift” model where the correct mean drift rate parameter (i.e., *v*_*c*_) was allowed to have different values across conditions, (3) a “threshold” model where the threshold parameter (i.e., *b*) was allowed to have different values between conditions, and (4) a “complex” model, where both *v*_*c*_ and *b* were allowed to have different values across conditions. Formally, the null model was defined as:

$$\begin{array}{@{}rcl@{}} v_{c} & \sim & TN(3,3,0,Inf) \\ v_{e} & \sim & TN(2,3,0,Inf) \\ sv_{e} & \sim & TN(2,3,0,Inf) \\ A & \sim & TN(2,2,0,Inf) \\ b - A & \sim & TN(2,2,0,Inf) \\ t_{0} & \sim & TN(0.5,0.5,0,Inf) \\ \end{array} $$

the drift model was defined as:

$$\begin{array}{@{}rcl@{}} v_{c,i} & \sim & TN(3,3,0,Inf) \\ v_{e} & \sim & TN(2,3,0,Inf) \\ sv_{e} & \sim & TN(2,3,0,Inf) \\ A & \sim & TN(2,2,0,Inf) \\ b - A & \sim & TN(2,2,0,Inf) \\ t_{0} & \sim & TN(0.5,0.5,0,Inf) \\ \end{array} $$

where *i* indexes the condition, the threshold model was defined as:

$$\begin{array}{@{}rcl@{}} v_{c} & \sim & TN(3,3,0,Inf) \\ v_{e} & \sim & TN(2,3,0,Inf) \\ sv_{e} & \sim & TN(2,3,0,Inf) \\ A & \sim & TN(2,2,0,Inf) \\ b_{i} - A & \sim & TN(2,2,0,Inf) \\ t_{0} & \sim & TN(0.5,0.5,0,Inf) \\ \end{array} $$

and the complex model was defined as:

$$\begin{array}{@{}rcl@{}} v_{c,i} & \sim & TN(3,3,0,Inf) \\ v_{e} & \sim & TN(2,3,0,Inf) \\ sv_{e} & \sim & TN(2,3,0,Inf) \\ A & \sim & TN(2,2,0,Inf) \\ b_{i} - A & \sim & TN(2,2,0,Inf) \\ t_{0} & \sim & TN(0.5,0.5,0,Inf) \\ \end{array} $$

### Model selection methods

I compared the inferences of nine different model selection methods, which have all been previously suggested or used for deciding between cognitive models. The first four methods are the commonly implemented “information criteria” discussed in the introduction: the Akaike Information Criterion (AIC; Akaike, 1974), the Bayesian Information Criterion (BIC; Schwarz, 1978), the Deviance Information Criterion (DIC; Spiegelhalter et al., 2002), and the Widely Applicable Information Criterion (WAIC; Vehtari et al., 2017). I also included an alteration on the standard DIC method that has been implemented previously within the literature (Osth, Dennis, & Heathcote, 2017), where the prior probability is included in the deviance calculations, meaning that the deviance is calculated based on the unnormalized posterior probability rather than the model likelihood.^1^ In addition, I included two recently proposed methods of calculating Bayes factors in cognitive models, which have been shown to provide an accurate approximation in simpler models where the integral is solvable (Xie, Lewis, Fan, Kuo, & Chen, 2010; Friel, Hurn, & Wyse, 2014; Friel & Wyse, 2012; Liu et al., 2016; Gronau et al., 2017): Bayes factors using Bridge Sampling (BF-BS; Gronau et al., 2017), and Bayes factors using Thermodynamic Integration (BF-TI; Annis et al., 2018) through the Thermodynamic Integration via Differential Evolution method (TIDE; Evans & Annis, 2019). As a parameter estimation-based method, I included an augmentation of the Region Of Practical Equivalence (ROPE; Kruschke, 2011; Kruschke & Liddell, 2018) method, which selects the region (i.e., either “practically zero”, or “not zero”) with the most posterior density, stopping the method from having indeterminate cases. Lastly, I included the well-known *χ*^2^ test of relative deviance, which is a simple maximum likelihood-based method.

The AIC, DIC, and WAIC each aim to provide an approximation of predictive accuracy, being asymptotically equivalent to leave-one-out cross validation (LOO-CV). AIC uses the maximum likelihood as a measure of goodness-of-fit, and the number of free parameters as a measure of flexibility, with more parameters resulting in harsher penalties. DIC uses the average log-likelihood over the posterior distribution as a measure of goodness-of-fit, and the difference between this average and the log-likelihood at some fixed, central point of the posterior as a measure of flexibility, with greater differences resulting in harsher penalties. Although the mean of the parameter values over the joint posterior is often used as the point estimate in this calculation (Spiegelhalter et al., 2002), I instead use the point of minimum deviance in the posterior for the point estimate (also recommended by Spiegelhalter et al., 2002), as the use of the mean results in the strong assumption that the joint posterior distribution is a multivariate normal, and can result in negative estimates of flexibility when this assumption is violated (Vehtari et al., 2017). WAIC uses a similar measure of goodness-of-fit as DIC, being the log of the average posterior likelihood for each data point, but uses the variance in log-likelihood over the posterior distribution as a measure of flexibility, with greater variances resulting in harsher penalties.

Formally, AIC can be written as:

$$\begin{array}{@{}rcl@{}} \hat{L} &=& \log[p(y|\boldsymbol{\theta}_{\max})] \\ P_{AIC} &=& k \\ AIC &=& -2(\hat{L}-P_{AIC}) \end{array} $$

where log[] is the natural logarithm function, *y* is the data, ***𝜃*** are the parameters, ***𝜃***_max_ are the parameter values that give that maximum likelihood (i.e., maximize the function *p*(*y*|*𝜃*)), and *k* is the number of free parameters in the model. DIC can be written as:

$$\begin{array}{@{}rcl@{}} \bar{D} &=& \frac{1}{S} \sum\limits_{s = 1}^{S} \log[p(y|\boldsymbol{\theta}_{s})] \\ P_{D} &=& \max[\log[p(y|\boldsymbol{\theta})]] - \bar{D} \\ DIC &=& -2(\bar{D}-P_{D}) \end{array} $$

where *S* is the number of posterior samples, and max[] is the maximum function over all posterior samples. WAIC can be written as:

$$\begin{array}{@{}rcl@{}} lpd &=& \sum\limits_{i = 1}^{n}\log\left[\frac{1}{S} \sum\limits_{s = 1}^{S} p(y_{i}|\boldsymbol{\theta}_{s})\right] \\ P_{waic} &=& \sum\limits_{i = 1}^{n} var[\log[p(y_{i}|\boldsymbol{\theta})]] \\ WAIC &=& -2(lpd-P_{waic}) \end{array} $$

where *n* is the number of data points, and *var*[] is the variance function over the posterior samples.

The BIC, BF-BS, and BF-TI each aim to provide an approximation of the Bayes factor. BIC uses the maximum likelihood as a measure of goodness-of-fit, and the number of free parameters combined with the number of observations as a measure of flexibility, with more parameters resulting in harsher penalties, and the relative penalty between models increasing as the number of observations increases. Both “BF” methods have no explicit measures of goodness-of-fit or flexibility, with the marginal likelihood (the Bayes factor is a ratio of the marginal likelihoods of each model) containing a natural penalty for flexibility through the integration of the unnormalized posterior probability over the entire parameter space. BF-BS uses the bridge sampling algorithm to estimate the marginal likelihood for each model, requiring (1) samples from the posterior distribution, (2) the definition of a proposal distribution and samples from this distribution, and (3) the definition of a bridge function. I use the recommendations of Gronau et al., (2017) for the proposal distribution and bridge function, defining the proposal distribution using the mean and variance of the estimated posteriors, and the optimal bridge function of Meng and Wong (1996). BF-TI uses the TIDE method to estimate the marginal likelihood for each model, requiring the estimation of the mean log-likelihood for several “power posteriors”. A power posterior is a posterior distribution estimated with the likelihood placed to a specific power (i.e., *p*(*y*|***𝜃***)^*t*^). To obtain the marginal likelihood, a series of power posteriors must be estimated with powers (known as “temperatures”; *t*) between 0 and 1, which forms a discrete approximation of a continuous integration curve between 0 and 1. Integrating over this area with a simple integration rule provides the marginal likelihood. The TIDE method estimates all power posteriors simultaneously, with each temperature being a different chain in a single run of the Different Evolution Markov chain Monte Carlo (DE-MCMC; Ter Braak, 2006; Turner, Sederberg, Brown, & Steyvers, 2013) sampling algorithm.

Formally, BIC can be written as:

$$\begin{array}{@{}rcl@{}} \hat{L} &=& \log[p(y|\boldsymbol{\theta}_{\max})] \\ P_{BIC} &=& \log[n]k \\ BIC &=& -2(\hat{L}-\frac{1}{2}P_{BIC}) \end{array} $$

and the extensive formal definitions for bridge sampling and thermodynamic integration can be seen in Gronau et al., (2017) and Annis et al., (2018), respectively.

The other three methods, DIC with the prior probability included (DIC_*p*_), the augmented ROPE (ROPE_*a*_), and the *χ*^2^ test of relative deviance, do not clearly fit into either category above. DIC_*p*_ is calculated in an identical manner to DIC, but with the deviance calculation based on the unnormalized posterior probability. ROPE_*a*_ is calculated in the same fashion as regular ROPE, where the selection process is purely based on the parameter posterior densities; however, in cases that would usually be ruled as “indeterminate”, ROPE_*a*_ chooses based on the largest posterior density. Note that as with ROPE, ROPE_*a*_ makes decisions on parameters as separate, binary cases. Therefore, to select the model with both drift rate and threshold varying over condition, ROPE_*a*_ would require the posterior distributions for the change in drift rate and threshold over condition to each mostly fall outside their respective ROPE. The *χ*^2^ method uses a significance test to decide whether additional parameters are required to explain the data in the case of nested models. Specifically, the difference in deviance—with the “deviance” in this case being the maximum log-likelihood multiplied by negative 2—is approximately distributed as a *χ*^2^ with degrees of freedom equal to the difference in the number of parameters. Significant values indicate that the more complex model is required, whereas non-significant values indicate that the simpler model is satisfactory. In order to use the *χ*^2^ test to compare all four models, I firstly assessed which “single effect” model (i.e., drift only or threshold only) had the better deviance score (as they have an equal number of parameters), and then compared the better model to the null model. If this test was significant, then the single effect model was compared to the drift and threshold model to determine the best model, and if the test was non-significant, then the null model was compared to the complex model to determine the best model.

Formally, DIC_*p*_ can be written as:

$$\begin{array}{@{}rcl@{}} \bar{D} &=& \frac{1}{S} \sum\limits_{s = 1}^{S} \log[p(\boldsymbol{\theta}_{s}|y)] \\ P_{D} &=& \max[\log[p(\boldsymbol{\theta}|y)]] - \bar{D} \\ DIC_{p} &=& -2(\bar{D}-P_{D}) \end{array} $$

where max[] is the maximum function over all posterior samples.

### Model fitting

All models were fit using Bayesian parameter estimation, as most of the model selection metrics assessed in this study require posterior distributions. The posterior distributions were estimated through Differential Evolution Markov chain Monte Carlo (DE-MCMC; Ter Braak, 2006; Turner et al., 2013), with 3*k* parallel chains (with *k* being the number of free parameters in the model), 1,000 samples per chain discarded as burn-in, and 1000 samples per chain taken from the joint posterior. For AIC, BIC, and the *χ*^2^ test of relative deviance, which all require the maximum likelihood, I used the maximum likelihood contained within the posterior distribution (i.e., *p*(*y*|***𝜃***)), which is equivalent to the maximum likelihood obtained through maximum likelihood estimation in situations where data are plentiful and the prior is relatively uninformative. For BF-BS, I took 3*k*× 1000 samples from the proposal distribution. For BF-TI, I fit each model with 40 temperatures, meaning that 40 chains were used instead of 3*k*. For ROPE_*a*_, the same value used to define a “small” effect size for each parameter in simulating the data was used to define the size of the ROPE (i.e., *v*_*c*_ = [− 0.3,0.3];*b* = [− 0.11,0.11]).

### Assessments of performance

I assessed the performance of the nine model selection methods in three different ways: the selection of the “correct” model, the selection of specific effects (i.e., drift or threshold), and the consistent between metrics. Note that I say “correct” (i.e., in inverted commas), as although the data generating model is known, the data generating model is not necessarily the model that best explains each specific sample of randomly generated data. Therefore, assessing the selection of the correct model is strictly an “inversion” based test (see Lee, 2018 for an explanation of the differences between inference and inversion).

The “selection of the correct model” assessment focused on how often the methods selected the data generating model as the best model, and the “selection of specific effects” focused on how often the methods selected each of the data generating effects in the best model, with the latter providing a more in-depth assessment of the former. These assessments were both performed according to two criteria: the proportion of correct selections in the 100 simulation in each cell of the design, and the average Brier score (Brier, 1950) over the 100 simulations. The Brier score is a scoring method that provides a measure of accuracy for probabilistic choices. Specifically, I used the original definition by Brier (1950):

$$\begin{array}{@{}rcl@{}} \text{Brier Score} = \frac{1}{R}\sum\limits_{i = 1}^{R} (f_{i} - o_{i})^{2} \end{array} $$

where *R* is the number of possible outcomes, *f*_*i*_ is the predicted probability for outcome *i*, and *o*_*i*_ is a binary variable for whether outcome *i* occurred (1 if it did, 0 if it did not). All possible Brier scores fall between 0 and 2, where 0 indicates the best possible performance and 2 indicates the worst possible performance. However, I adjusted the resulting Brier scores to a scale that provides a more intuitive interpretation, where scores are between − 1 and 1, − 1 indicates the worst possible performance, 1 indicates the best possible performance, and 0 indicates chance performance. In order to obtain the probability assigned to each outcome for each model in a specific data set, I calculated probability weights for each model. For models on the deviance scale (i.e., AIC, BIC, DIC, WAIC, and DIC_*p*_), the probability weight for model *i* was calculated as:

$$\begin{array}{@{}rcl@{}} W_{i} = \frac{exp(MSM_{i} \times (-0.5))}{{\sum}^{N}_{j = 1} exp(MSM_{j} \times (-0.5))} \end{array} $$

where *MSM*_*i*_ is the model selection method deviance for model *i*, and N in the number of models being evaluated. For models on the log-likelihood scale (i.e., BF-TI and BF-BS) the probability weight for model *i* was calculated as:

$$\begin{array}{@{}rcl@{}} W_{i} = \frac{exp(MSM_{i})}{{\sum}^{N}_{j = 1} exp(MSM_{j})} \end{array} $$

For ROPE_*a*_, the same concept was used as above, except instead of a likelihood being used to calculate the weights, it was the relative posterior density. However, the Brier score cannot be easily calculated for the *χ*^2^ test of relative deviance, as the choice is determined through a significance test, which cannot be directly interpreted as a level of evidence (Wagenmakers, 2007). Therefore, the *χ*^2^ test of relative deviance was excluded from the Brier score assessment.

The final assessment, the “consistency between metrics”, focused on how often the different methods agree in their chosen model. Specifically, for each replicate in each cell of the design, I assess the proportion of instances where the methods selected the same model, for each pairing of methods. Note that this is the consistency in the joint selections (i.e., out of the four possible models), and not the consistency between the selection of separate drift and threshold effects.

## Simulation study

### Simulations

I simulated 2500 total data sets, with each being a single simulated “participant” with two “experimental” conditions, and 300 simulated trials per condition. My choice of two experimental conditions, rather than a larger number, was to (1) minimize the potential sources of variability in the design to allow comparisons to be as robust as possible, and (2) mimic the pseudo-experiment design of Dutilh et al., (2018), which my study intends to provide a follow-up to. My choice of 300 trials per conditions was based around this approximately reflecting the number of rapid decision (i.e., 600) that participants often make in short rapid decision-making experiments (e.g., Trueblood, Brown, Heathcote, & Busemeyer, 2013; Hawkins et al., 2014a, b; Evans & Brown, 2017; Evans et al., 2017; Evans, Hawkins, Boehm, Wagenmakers, & Brown, 2017).

The 2500 data sets fell into one of five different qualitative types of effects: “null”, “drift”, “threshold”, “drift and threshold extreme”, and “drift and threshold balanced”. The “null” data sets were simulated using the same parameter values for both conditions, meaning that there was no underlying difference between the two conditions. The “drift” and “threshold” data sets were simulated with an increased (decreased) drift rate (threshold) parameter value for the second condition, respectively. The amount of change in the parameter for each data set was set to be either “small”, “moderate”, or “large”, with these different sizes decided by a priori simulations that found what amount of change resulted in 0.2, 0.5, and 0.8 effect size differences between conditions in the response time distributions. This created six different categories of one-way effects: drift rate and threshold, with each having small, moderate, or large differences in the parameter value. The “drift and threshold extreme” data sets were simulated using an increase in drift rate *and* a decrease in threshold for the *same* condition, creating a more *extreme* difference between the conditions in response time than a single effect. The “drift and threshold balanced” data sets were using an increase in drift rate for one condition, and a decrease in threshold for the other condition (i.e., *different* conditions), with the effects in response time *balancing* each other out to some extent.^2^ Combining small, moderate, and large effects in drift rate and threshold factorially, for extreme and balanced effects, created 18 different categories of two-way effects, making 25 categories of effects in total.

I simulated 100 “participants” (i.e., data sets) for each of these 25 categories of effects, which served as replicates of the effects to assess the consistency of inferences. To minimize the potential sources of variability that could causes inconsistencies, these simulated replicates were each generated with identical parameter values. In addition, across the 25 categories of effect, the same parameter values were used for non-manipulated parameters. These “baseline”, non-manipulated parameter values were:

$$\begin{array}{@{}rcl@{}} v_{c} = 3 \\ v_{e} = 2 \\ sv_{c} = 1 \\ sv_{e} = 1 \\ A = 1 \\ b = 2 \\ t_{0} = 0.3 \\ \end{array} $$

and drift rate and threshold were changed to the following values when the effect was used to generate the data set:

$$\begin{array}{@{}rcl@{}} & v_{c} & b \\ \text{Small} & 3.3 & 1.89 \\ \text{Moderate} & 3.75 & 1.74 \\ \text{Large} & 4.25 & 1.595 \\ \end{array} $$

### Results

#### Selection of correct model

**Fig. 1:**
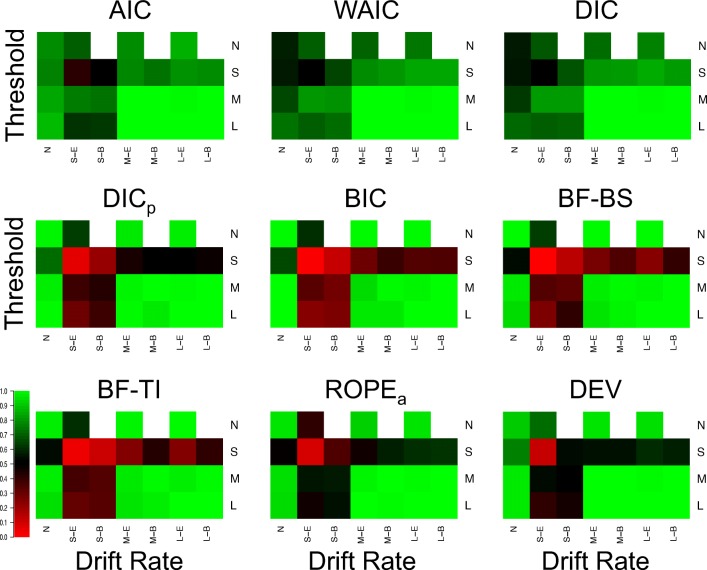
Plots of the proportion of correct selections for each model selection method (different plots) for the 25 different cells of the design (rows and columns). *Lighter shades of green* indicate better performance, *lighter shades of red* indicate worse performance, and *black* indicates intermediate performance, which can be seen in the color bar to the left-hand side. *White* indicates cells that did not exist in the simulated design. Different cells display different data-generating models, with the different columns being different generated drift rates, and the different rows being different generated thresholds. For rows and columns, ‘N’ refers to no effect, ‘S’ refers to a small effect, ‘M’ refers to a moderate effect, and ‘L’ refers to a large effect. When both effects are present (i.e., not ‘N’), ‘E’ refers to an extreme difference between conditions, whereas ‘B’ refers to a balanced difference between conditions

**Fig. 2:**
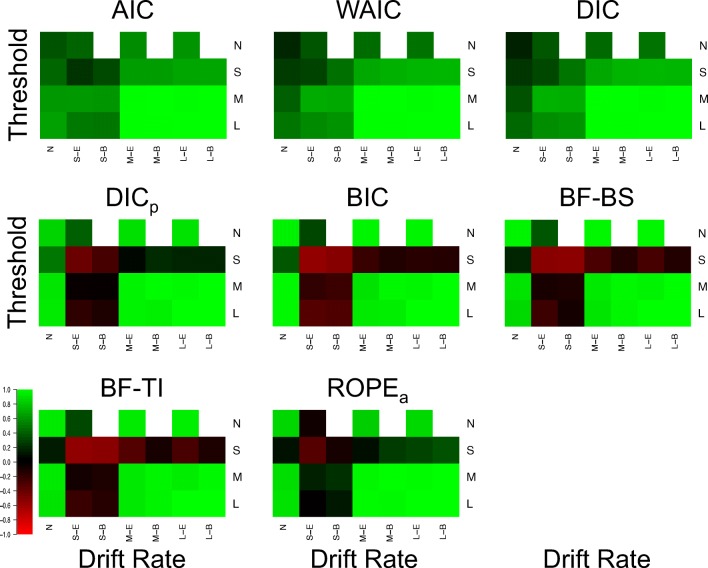
Plots of the Brier scores of correct selections for each model selection method (different plots) for the 25 different cells of the design (rows and columns). *Lighter shades of green* indicate better performance, *lighter shades of red* indicate worse performance, and *black* indicates intermediate performance, which can be seen in the color bar to the left-hand side. *White* indicates cells that did not exist in the simulated design. Different cells display different data-generating models, with the different columns being different generated drift rates, and the different rows being different generated thresholds. For rows and columns, ‘N’ refers to no effect, ‘S’ refers to a small effect, ‘M’ refers to a moderate effect, and ‘L’ refers to a large effect. When both effects are present (i.e., not ‘N’), ‘E’ refers to an extreme difference between conditions, whereas ‘B’ refers to a balanced difference between conditions

Figure 1 displays the proportion of correct selections for each model selection method (different plots) in each of the 25 different cells of the simulated design, and Fig. 2 displays the average Brier scores.^3^ In general, the Brier scores lead to the same conclusions as the proportion of correct selections throughout the entire study, and so these different criteria will not be discussed separately. However, one important difference to note is that the performance for all methods appears to be better (i.e., closer to the best possible performance) for the Brier score criterion than the proportion of correct selections. This suggests that when the methods select the correct model, they do so with greater confidence (i.e., higher probability) than when they select the incorrect model, meaning that the probability assigned to each model provides important information beyond the discrete selection of the best model.

There appear to be two overarching trends in the correct selections. Firstly, all methods correctly identify almost every replicate when both effects are part of the data generating model and each effect is either moderate or large. This suggests that (1) the methods are indistinguishable in these circumstances, and (2) by inversion standards, all methods show near-optimal performance in these circumstances. Secondly, the performance of the methods appears to be clearly separated in the small effect and null data sets. In general, AIC, DIC, and WAIC appear to show good performance in each of these cells of the design, whereas BIC, BF-BS, BF-TI, DIC_*p*_, and ROPE_*a*_ show near-optimal performance when either no effect is present (i.e., the null data set) or a single moderate/large effect is present, but show poor performance when one or both of the effects are small. This suggests that (1) no method appears to perform “better” than all others under all potential conditions, and (2) some methods are more consistent in their selection accuracy across the different potential combinations of effects, whereas others are more polarizing.

In addition to these general trends, there appear to be three other important, and potentially surprising, more specific results. Firstly, when including the prior probability, the DIC method (i.e., DIC_*p*_) has a similar pattern of performance across the cells of the design to the Bayes factor methods, as opposed to the predictive accuracy methods that the standard DIC method belongs to. The performance of DIC and DIC_*p*_ deviate from one another a great deal, suggesting a large influence of including the prior probability. Secondly, the conceptually simple ROPE_*a*_ method performs very well, even though augmentations were made to the standard ROPE method to force it to select a model in every instance. In general, the augmented ROPE seems to perform as well as, if not better than, the Bayes factor. Lastly, and perhaps most importantly, the simpler parameter-counting methods perform extremely well. In each cell of the design, AIC performs about as well as both of its more complex counterparts, DIC and WAIC, and BIC performs about as well as its more complex Bayes factor counterparts. This is extremely important, as the use of more complex metrics is commonly advocated on theoretical bases, but in this practical context, their performance does not seem to be any better than the simpler methods. The simple *χ*^2^ deviance test (DEV) also shows good performance, though it does not seem to clearly map onto either class of selection method.

#### Selection of specific effects

**Fig. 3:**
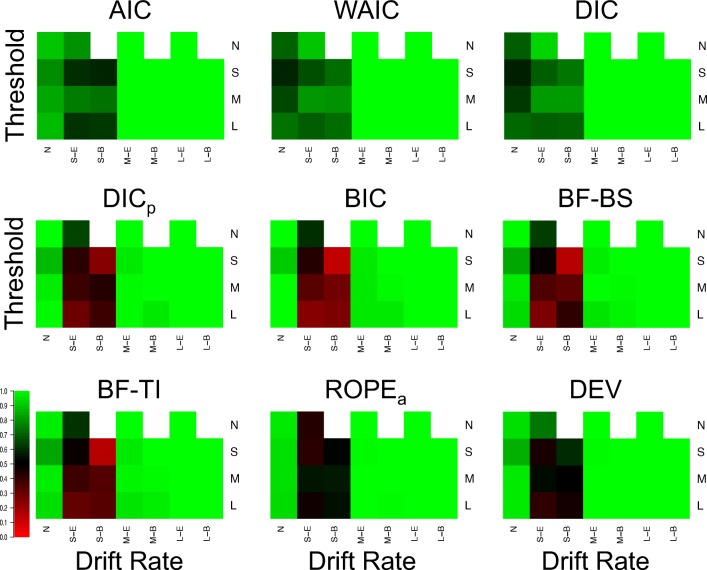
Plots of the proportion of correct selections for the drift rate effect for each model selection method (different plots) for the 25 different cells of the design (rows and columns). *Lighter shades of green* indicate better performance, *lighter shades of red* indicate worse performance, and *black* indicates intermediate performance, which can be seen in the color bar to the left-hand side. *White* indicates cells that did not exist in the simulated design. Different cells display different data-generating models, with the different columns being different generated drift rates, and the different rows being different generated thresholds. For rows and columns, ‘N’ refers to no effect, ‘S’ refers to a small effect, ‘M’ refers to a moderate effect, and ‘L’ refers to a large effect. When both effects are present (i.e., not ‘N’), ‘E’ refers to an extreme difference between conditions, whereas ‘B’ refers to a balanced difference between conditions

**Fig. 4:**
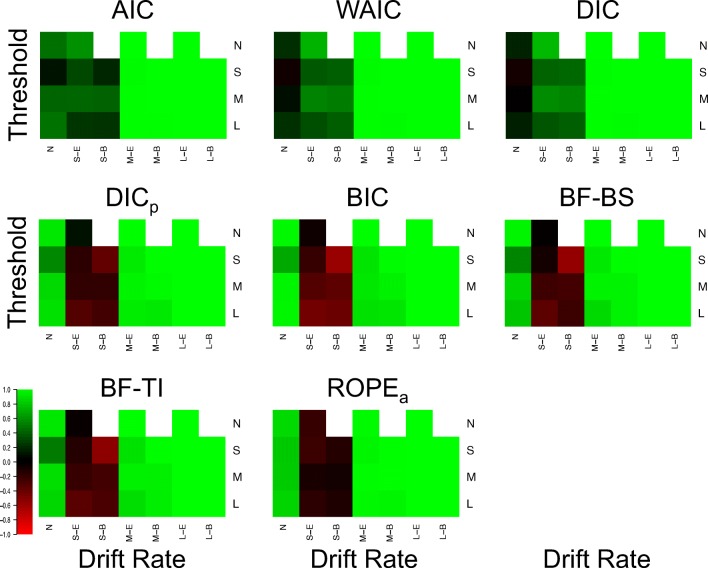
Plots of the Brier score of correct selections for the drift rate effect for each model selection method (different plots) for the 25 different cells of the design (rows and columns). *Lighter shades of green* indicate better performance, *lighter shades of red* indicate worse performance, and *black* indicates intermediate performance, which can be seen in the color bar to the left-hand side. *White* indicates cells that did not exist in the simulated design. Different cells display different data-generating models, with the different columns being different generated drift rates, and the different rows being different generated thresholds. For rows and columns, ‘n refers to no effect, ‘S’ refers to a small effect, ‘M’ refers to a moderate effect, and ‘L’ refers to a large effect. When both effects are present (i.e., not ‘N’), ‘E’ refers to an extreme difference between conditions, whereas ‘n refers to a balanced difference between conditions

**Fig. 5:**
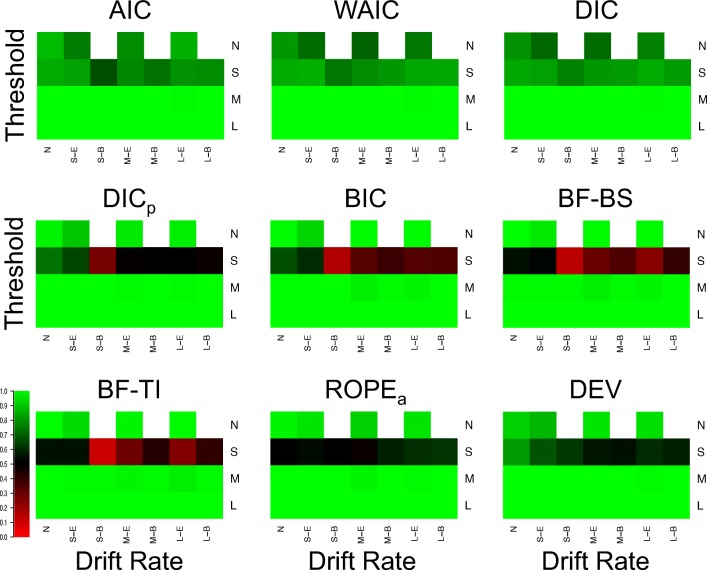
Plots of the proportion of correct selections for the threshold effect for each model selection method (different plots) for the 25 different cells of the design (rows and columns). *Lighter shades of green* indicate better performance, *lighter shades of red* indicate worse performance, and *black* indicates intermediate performance, which can be seen in the color bar to the left-hand side. *White* indicates cells that did not exist in the simulated design. Different cells display different data-generating models, with the different columns being different generated drift rates, and the different rows being different generated thresholds. For rows and columns, ‘N’ refers to no effect, ‘S’ refers to a small effect, ‘M’ refers to a moderate effect, and ‘L’ refers to a large effect. When both effects are present (i.e., not ‘N’), ‘E’ refers to an extreme difference between conditions, whereas ‘B’ refers to a balanced difference between conditions

**Fig. 6:**
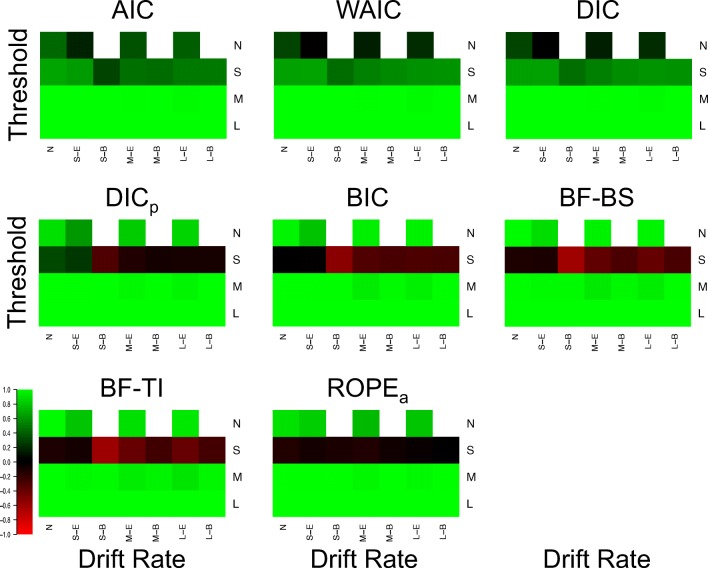
Plots of the proportion of correct selections for the threshold effect for each model selection method (different plots) for the 25 different cells of the design (rows and columns). *Lighter shades of green* indicate better performance, *lighter shades of red* indicate worse performance, and *black* indicates intermediate performance, which can be seen in the color bar to the left-hand side. *White* indicates cells that did not exist in the simulated design. Different cells display different data-generating models, with the different columns being different generated drift rates, and the different rows being different generated thresholds. For rows and columns, ‘N’ refers to no effect, ‘S’ refers to a small effect, ‘M’ refers to a moderate effect, and ‘L’ refers to a large effect. When both effects are present (i.e., not ‘N’), ‘E’ refers to an extreme difference between conditions, whereas ‘B’ refers to a balanced difference between conditions

Figure 3 displays the proportion of correct drift selections for each model selection method in each of the 25 different cells (Fig. 4 for the Brier scores), and Fig. 5 displays the same for threshold selections (Fig. 6 for the Brier scores). As the previous section already displayed near-optimal performance for all methods in data sets where both effects were present and moderate or large in size, showing that all methods almost always select both drift and threshold effects in these situations, the focus of this section will be on the other, more distinguishing cells of the design.

There appear to be several key trends in the effect selection. Firstly, there appears to be little-to-no difference in how the methods identify different types of effects (i.e., drift or threshold) of similar effect sizes, with drift and threshold being selected fairly similar amounts in equivalent cell of the design. Secondly, the “Bayes factor” type methods are much more conservative than the “predictive accuracy” type methods. The clear distinction here is in cases where the effect does not exist (first column of Fig. 3, and first row of Fig. 5), or is small (second and third columns of Fig. 3, and second row of Fig. 5). In each of these cases, BIC, BF-BS, BF-TI, ROPE, and DIC_*p*_ rarely select the effect, in contrast to AIC, DIC, and WAIC, which more commonly select the effect. This results in AIC, DIC, and WAIC commonly suggesting that an effect exists when it does not, but also results in BIC, BF-BS, BF-TI, ROPE, and DIC_*p*_ often suggesting that no effect exists when it does, but is small. This finding probably isn’t surprising given that the “predictive accuracy” methods do not aim to identify the true model and asymptotically select the more flexible model, and Bayes factors with uninformed priors are known to be conservative. However, it is interesting to note that this conservative nature only makes a practical difference when the effect is small or non-existent.

Thirdly, the selection of one effect (e.g., drift rate) appears to be largely independent of whether or not the other effect (e.g., threshold) is non-existent, small, moderate, or large. For example, when looking at Fig. 3, the number of selections of a drift rate effect when it exists and is small (columns 2 and 3) appears to differ little based on whether the threshold effect is small (row 2), moderate (row 3), or large (row 4). Lastly, when both effects are present and small, whether the effects are “extreme” (i.e., drift rate increasing and threshold decreasing in the same condition) or “balanced” (i.e., drift rate increasing and threshold decreasing in different conditions) appears to have some impact on the identification of each effect. Specifically, DIC_*p*_, BF-BS, BF-TI, and BIC all seem to show fewer selection of each effect when the effects are “balanced”, compared to when they are “extreme”, suggesting that two opposing effects that push the response time distributions to be similar to one another may be difficult to identify. However, it should be noted that this trend is smaller and less consistent across methods than the other trends discussed.

#### Consistency between metrics

**Fig. 7:**
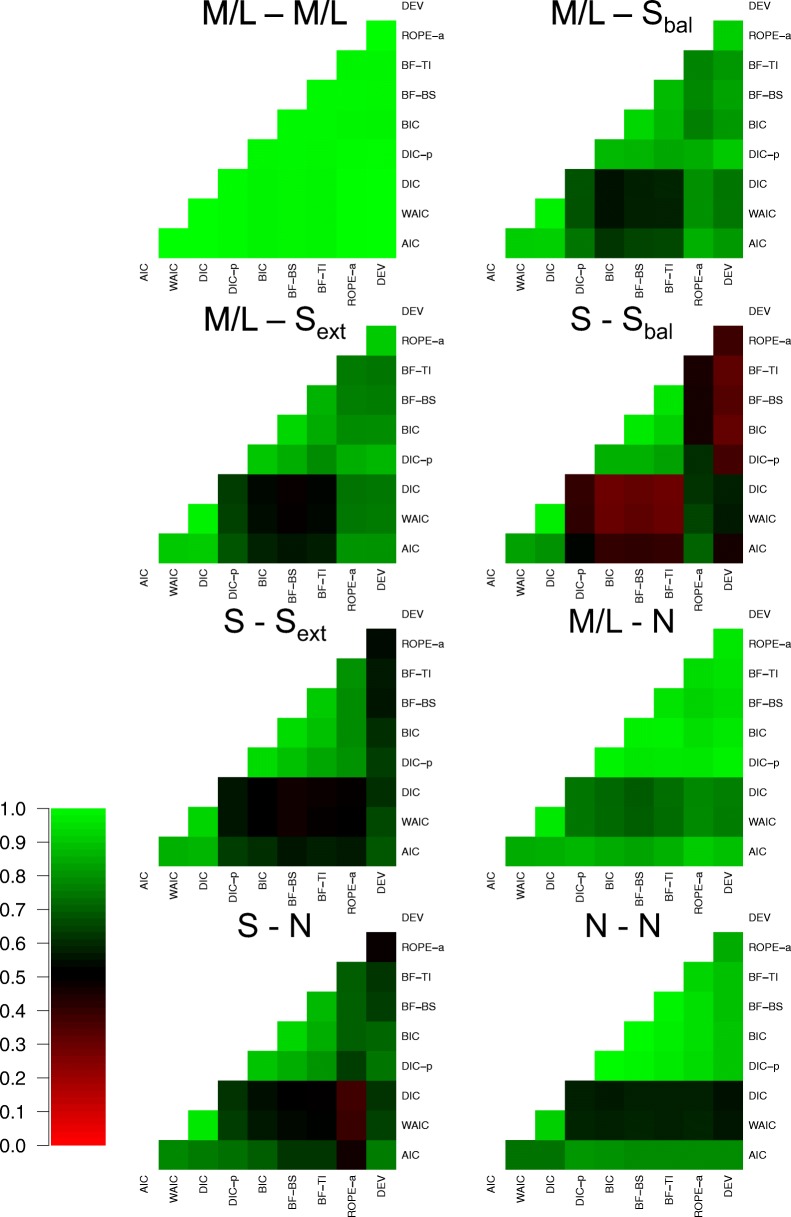
Plots the agreement in selected model between each of the eight model selection methods (rows and columns of each plot) for eight different groupings of the data (different plots). *Lighter shades of green* indicate greater agreement, *lighter shades of red* indicate greater disagreement, and *black* indicates intermediate agreement, which can be seen in the color bar to the left-hand side. For the groupings of the data, ‘n refers to no effect, ‘S’ refers to a small effect, and ‘M/L’ refers to a moderate or large effect. The two different letters refer to whether the data were generated with both effects, one effect, or neither effect. When the data were generated with both effects, the subscript ‘bal’ refers to a balanced difference between conditions, and the subscript ‘ext’ refers to an extreme difference between conditions

As the relationship between methods was extremely similar in many cells of the design, I collapsed over several for ease of communication. Firstly, as there was little difference between the methods in previous assessments when both effects were present and moderate or large in size, I collapse over these 8 cells. Secondly, as there was generally little difference between the impact of moderate and large effects in previous assessments, I collapse over those cells in all instances. Lastly, as there was little difference between drift rate and threshold of effects in previous assessments, I collapse over these two types of generated effects. This leaves eight collapsed cells to assess the consistency of the methods over, which can be seen in Fig. 7.

There appear to be two key general trends in the consistency assessment. First and foremost, all methods are extremely consistent with one another when both effects are present and moderate-to-large in size. This further re-iterates that the choice in model selection method makes little difference in situations with sizable effects. Secondly, there appears to become a much greater distinction between the predictive accuracy and Bayes factor methods in the other cells of the design, where one or both of the effects are small or non-existent. The difference appears to be least prevalent when one effect is large and the other does not exist, and most prevalent when both effects exist, are small, and are “balanced”, further showing that the key distinction between the methods is for small effects. However, in all cases, the consistency within each class of method is very high, with AIC, DIC, and WAIC all being consistent with one another, and BIC, BF-BS, and BF-TI also being consistent with one another.

In addition, there appear to be two specific, but important, points to note here. Firstly, DIC and WAIC are extremely consistent with one another in every cell of the design. Although this may seem like a minor point, the lack of difference between these methods is of key importance. Specifically, WAIC has been suggested as a major theoretical improvement to DIC, and the assessment of the variance in the posterior likelihood is thought to be a superior assessment of flexibility than DIC’s more simple flexibility assessment. However, there appears to be little practical difference between the methods, suggesting that WAIC is of little-to-no benefit over the more simple DIC. Secondly, although the consistency between the two Bayes factor approximations of BF-BS and BF-TI is extremely high, they do not show perfect agreement. This is potentially concerning, as both methods are intended to estimate the exact same quantity (the marginal likelihood) in an unbiased manner, and have been shown to do so accurately in models where the marginal likelihood is analytically solvable (Xie et al., 2010; Friel et al., 2014; Friel & Wyse, 2012; Liu et al., 2016; Gronau et al., 2017). This suggests that the small number of posterior samples used to perform these assessments may have resulted in approximation error within one/both of these methods. Therefore, future research should aim to explore the sources of variability in these different methods.

This section also re-enforces several previous key points regarding the relationships between the different methods. Firstly, there appears to be a practical distinction between the “predictive accuracy” methods and the “Bayes factor” methods, with these classes of methods only providing largely consistent inferences when all effects evaluated exist and are sizable. This is very important to showcase, as researchers often use these different classes of methods interchangeably as general “model selection metrics”. However, the classes of methods are theoretically very different, and as shown here, can come to very different practical inferences. Secondly, DIC_*p*_ shows a much closer relationship to the “Bayes factor” methods than the “predictive accuracy” methods, including the standard DIC calculation, suggesting that the inclusion of the prior probability in the DIC calculation drastically changes its inferences, and makes it closer to an approximation of the Bayes factor (with uninformed priors) than the predictive accuracy. Thirdly, the augmented version of ROPE performed very similarly to the Bayes factor. However, ROPE_*a*_ was not always in perfect agreement with the Bayes factor, showing closer agreement to the predictive accuracy methods in the “small balanced” cell, which resulted in it having “better” performance than the Bayes factor in this cell. Lastly, the simpler methods of model selection showed a very close relationship to their more complex counterparts. In most cells of the design, AIC is extremely consistent with DIC and WAIC, and BIC is extremely consistent with the BF-BS and BF-TI. This suggests that although there are many theoretical advantages to using these more complex methods of model selection, the practical advantages of using them over more simple approximations may be limited, and therefore, many users may wish to opt for the simpler alternatives.

### Prior sensitivity assessment

One key finding of the simulation study was that both BIC and Bayes factors performed conservatively when compared to AIC, DIC, and WAIC. As mentioned above, this is probably not surprising given that Bayes factors with uninformative priors are known to be conservative. Specifically, when applying Bayes factors to simple, statistical models, previous research has shown that effects are less likely to be detected when uninformed priors are placed on the “effect size” parameter (i.e., the difference in condition means normalized by the standard deviation), compared to when using more informed priors (Lindley, 1957; Rouder, Morey, Speckman, & Province, 2012).

Here, I provide a follow-up assessment to evaluate how more informative priors practically impact the LBA models selected by the Bayes factor. As the Bayes factor provided near-optimal selection for moderate and large effects even with uninformative priors, I focus this assessment on the 5 cells of the design that are most likely to be impacted by using informed priors: the no effect, small drift rate effect, small threshold effect, small drift rate and threshold “extreme” effect, and small drift rate and threshold “balanced” effect. In order to place more informative priors on the difference in the parameter values between conditions,^4^ I re-parameterized the models that allowed differences between conditions to estimate the mean value of the parameter over conditions, and the difference in the parameter value between conditions, rather than the previous estimation of a parameter for each condition. Formally, this meant that the values for parameter *X* in conditions 1 and 2 were given by:

$$\begin{array}{@{}rcl@{}} X_{1} &=& X_{mean} - X_{diff} \\ X_{2} &=& X_{mean} + X_{diff} \end{array} $$

where *X*_*mean*_ is the parameter mean over conditions, and *X*_*diff*_ is the parameter difference between conditions. For the parameter reflecting the mean over conditions, I used the same priors as the previous analysis:

$$\begin{array}{@{}rcl@{}} v_{c} & \sim & TN(3,3,0,Inf) \\ b_{i} - A & \sim & TN(2,2,0,Inf) \end{array} $$

and for the parameter reflecting the difference between conditions, I defined four different levels of “informativeness” as different priors:

$$\begin{array}{@{}rcl@{}} UP & \sim & N(0,10) \\ WIP & \sim & N(0,1) \\ MIP & \sim & N(0,0.1) \\ HIP & \sim & N(0,0.01) \end{array} $$

where *UP* is a completely uninformative prior (even less informative than those used in the previous analysis), *WIP* is a weakly informative prior (slight more informative than those used in the previous analysis), *MIP* is a moderately informative prior, and *HIP* is a highly informative prior. As the focus of this assessment was only on the impact of informative priors, I only applied one of the methods (bridge sampling) of approximating the Bayes factor.

**Fig. 8:**
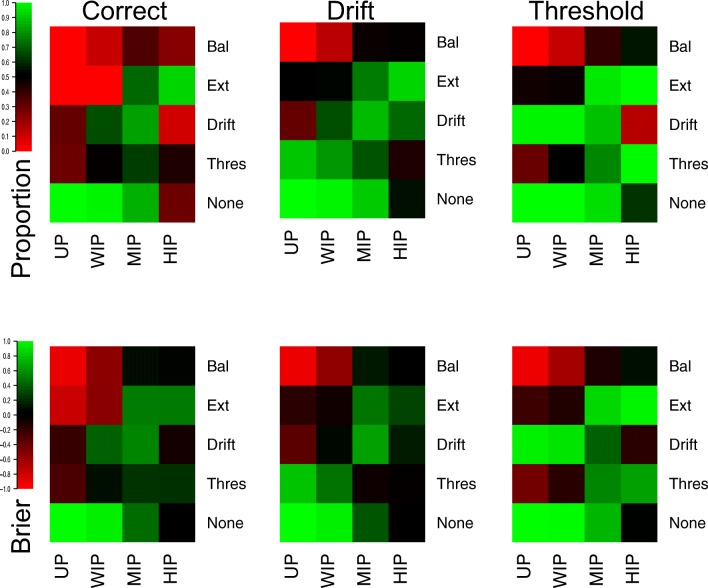
Plots of the correct (*left panels*), drift (*middle panels*), and threshold (*right panels*) selections, as proportions (*top panels*) and average Brier scores (*bottom panels*). *Lighter shades of green* indicate better performance, *lighter shades of red* indicate worse performance, and *black* indicates intermediate performance, which can be seen in the color bar to the left-hand side. Different rows of cells display different data-generating models, and different columns display different priors

Figure 8 displays the proportion of correct selections (top-left panel) for each prior in each of the 5 different cells of the design selected for this analysis, and the average Brier scores (bottom-left panel). The *WIP* shows extremely similar performance to the priors used in the previous analysis, showing near-optimal performance when no effect is present, poorer performance when a single, small sized effect is present, and extremely poor performance when both effects are present and small in size. The *UP* shows even more conservative performance, selecting optimally when no effect is present, but poorly for a single effect, and misidentifying almost every data set when both effects are present. In contrast, the *MIP* shows more balanced performance, with selection no longer being optimal when no effect is present, but drastically improving when either one or both effects are present. However, the *HIP* appears to become overly liberal, showing poorer performance in all cells of the design except the case where both effects are present and combined in an “extreme” manner.

The correct selection of specific effects (middle and right panels) supports the previous insights. The *UP* and *WIP* very rarely select an effect when it is not present, though often do not select effects that are present. The *MIP* performs better in this regard, mostly selecting effects when they are present and failing to select effects when they are not present, except for in the “balance” cell where selection is poorer. The *HIP* provides the opposite performance to the *UP* and *WIP*, often selecting effects regardless of whether they are present, except for in the “balance” cell, where selection is at approximately chance.

Importantly, these results show that the level of informativeness within the prior has a practical impact in the selection of psychological theory within the LBA when using Bayes factors, and using more informed priors can result in better performance. However, making these priors overly narrow (i.e., the *HIP* in this case) can have detrimental effects on the selection process, resulting in many effects that are not present being detected, and other strange patterns of selection (e.g., the “balance” cell of the design). Therefore, the use of informed priors can provide advantages for model selection with Bayes factors, though to provide sensible results these priors must be sensibly informed.

### Impact of sample size

As discussed at the beginning of the simulation study, I restricted my assessment to only a single number of trials per condition (300), which reflected an approximate number of rapid decisions that participants make in a short experiment. However, the number of trials can differ greatly between studies that apply EAMs, with some studies having as few as 40 trials per condition (e.g., Voss, Rothermund, & Voss, 2004) and others having thousands of trials per condition (e.g., Dutilh, Vandekerckhove, Tuerlinckx, & Wagenmakers, 2009). Importantly, different model selection methods can be impacted in different ways by the number of trials: for example, the flexibility penalty in BIC is dependent on the number of trials, whereas in AIC the flexibility penalty remains constant over differing numbers of trials.

Here, I provide a follow-up assessment to evaluate how differing numbers of trials per condition impact the selections made by the predictive accuracy and Bayes factor classes of methods. As both classes of methods showed near-optimal selection for moderate and large effects, I focus this assessment on the five cells of the design that provided the greatest distinction between the classes of methods. For each of these five cells, I simulated using four different numbers of trials per condition: 30, 100, 300 (i.e., the data sets from the previous assessment), and 900. As with the first simulation study, I simulated 100 data sets (i.e., “participants”) for each of these 20 cells. As there seemed to be general agreement within each class of methods in the previous simulation study, I only use four methods within this assessment: AIC (predictive accuracy, parameter counting), DIC (predictive accuracy, functional form), BIC (Bayes factor, parameter counting), and bridge sampling (Bayes factor, functional form; using the same priors as in the original simulation study results).

**Fig. 9:**
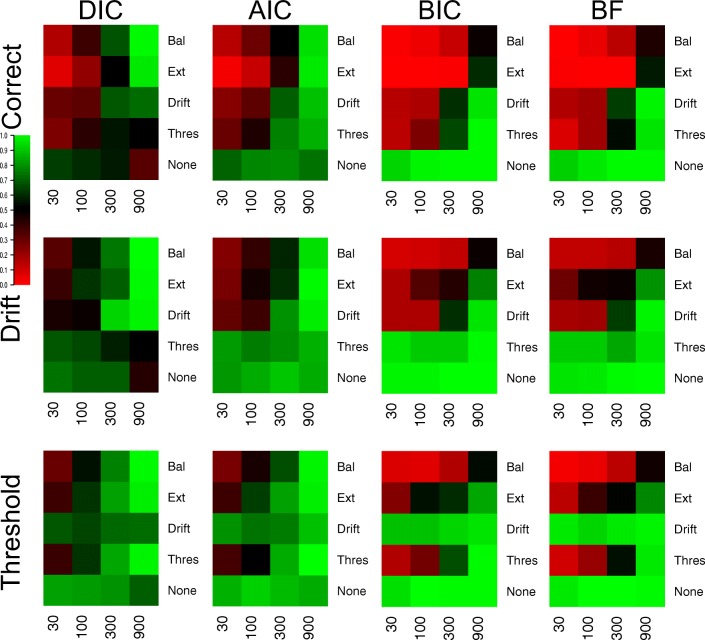
Plots of the proportion of correct (*top panels*), drift (*middle panels*), and threshold (*bottom panels*) selections for each model selection method (different columns of panels) for the 20 different cells of the design (rows and columns). *Lighter shades of green* indicate better performance, *lighter shades of red* indicate worse performance, and *black* indicates intermediate performance, which can be seen in the color bar to the left-hand side

**Fig. 10:**
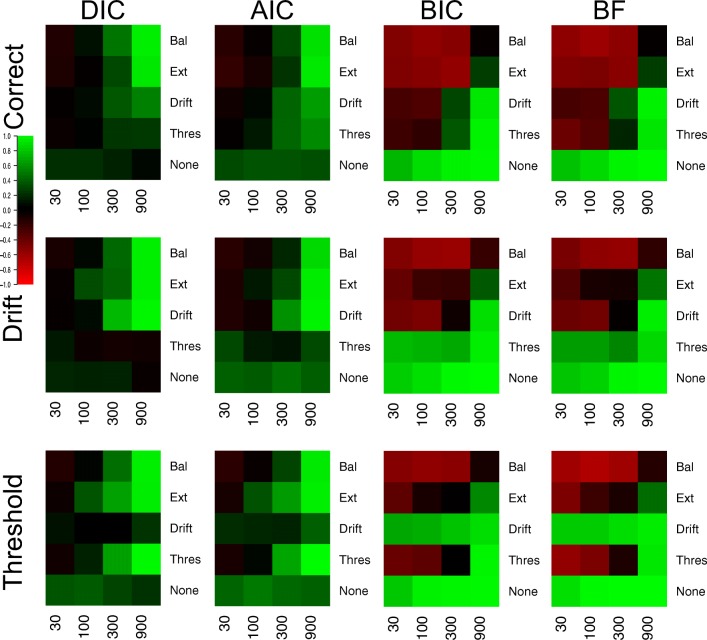
Plots of the Brier scores for the correct (*top panels*), drift (*middle panels*), and threshold (*bottom panels*) selections for each model selection method (different columns of panels) for the 20 different cells of the design (rows and columns). *Lighter shades of green* indicate better performance, *lighter shades of red* indicate worse performance, and *black* indicates intermediate performance, which can be seen in the color bar to the left-hand side

Figure 9 (top row) displays the proportion of collection selections for the different methods (different panels) across different cells of the design, and Fig. 10 (top row) displays the average Brier scores. For all methods the overall performance improves as sample size increases, especially within the cells of the design where effects are present. The middle and bottom panels of these figures show that this improvement is due to effects being selected less often for smaller sample sizes than larger sample sizes, with this trend continuing to largest number (900) of trials per condition, meaning that increasing the number of trials increases the “power” to detect these effects.

However, the performance of the different classes of methods in each cell of the design differed between the numbers of trials. For the smaller numbers of trials (30 and 100), the Bayes factor methods very rarely suggest that an effect is present, regardless of the true generating model. This results in near-perfect performance when no effect is present, but poor performance when either one or both of the effects are present. The predictive accuracy methods suggest that an effect is present slightly more often than the Bayes factor methods for smaller numbers of trials, resulting in slightly better performance when effects are present, and slightly poorer performance when they are not. However, it should be noted that at an absolute level, the predictive accuracy methods still perform poorly (i.e., below chance) in detecting these effects when the number of trials are small. For the largest number of trials (900), the predictive accuracy methods are near-perfect in detecting effects when they are present, resulting in near-perfect performance for the cells of the design where both effects are present. However, these methods also suggest that effects are present in some cases when they are not, resulting in poorer performance for the single effect and null cells. In contrast, the Bayes factor methods are near-perfect for the null and single effect cells, but fail to detect both effects in many cases for the “balance” and “extreme” data sets.

Interestingly, both Bayes factor methods show similar performance in all cells of the design, throughout all sample sizes. This again suggests that BIC provides an adequate approximation to the Bayes factor with uninformative priors for comparing different theories within the LBA framework. However, this is not the case for the predictive accuracy methods. Although AIC and DIC perform similarly when the number of trials are small, their performance continues to diverge as the number of trials increases. Specifically, as the number of trials increases, both AIC and DIC improve at detecting when effects are present, increasing their overall performance. However, as the number of trials increases AIC remains fairly stable at detecting when effects are not present, whereas DIC begins to false alarm increasingly often, resulting in DIC having poorer performance than AIC for single effect and null cells. This is likely due to DIC providing a more accurate approximation of leave-one-out cross validation than AIC. When using cross validation in cases where the null model is true, the estimated difference in parameter value between conditions in the more flexible model will become increasingly closer to zero (i.e., the fixed value of the null model) as the amount of data increases, which will result the more flexible model being identical to the simpler model in predicting the left out data (Gronau & Wagenmakers, 2018). However, as AIC contains an explicit flexibility penalty based on the number of free parameters in the model, the null model retains an advantage over the more flexible model in cases where the null model is true.

## Empirical study (data of Dutilh et al., 2018)

So far, I have assessed how these different model selection methods perform when compared to one another on simulated data, where the ground truth is known and is one of the models specified. These simulations provide a useful benchmark for assessing the performance of model selection methods, as the model selected by each method can be directly compared to the ground truth used to generate the data, allowing an assessment of how often the method makes the “correct” selection. However, these simulations are limited in a practical sense, as all models are likely to be wrong is some way (e.g., Box & Draper, 1987), and therefore, the ground truth within the simulations is unlikely to be the same one that exists within empirical data. Importantly, this model mis-specification could result in the comparisons from the previous simulated assessment not necessarily extrapolating to practical implementations of the LBA to empirical data. In addition, noise is often present in empirical data, due to factors such as measurement noise and contaminated responses, which can also make inferences more difficult (Evans & Brown, 2018).

In order to address the potential limitations of my simulated assessments, I performed the same assessments on the data of Dutilh et al., (2018). Note that within empirical data there is no objectively “correct” inference, but following Dutilh et al., I defined the “correct” inference as the one that follows the selective influence assumption: that is, difficulty manipulations only influence drift rate, and speed–accuracy instructions only influence threshold (though see Voss et al., 2004 and Rae et al., 2014 for potential problems with this assumption). However, I believe that less weight should be placed on the identification of the “correct” effect in these situations, and more weight should be placed on what inferences are made in each situation and the consistency between methods. Importantly, the latter assessments will allow some insight into whether the conclusions from the simulated study hold for empirical data.

A full explanation of the (Dutilh et al., 2018) experiment can be found in Dutilh et al.,, though I will provide a brief outline here. Twenty participants completed 18 blocks of a random dot motion task, each with 156 trials, which required participants to judge whether the general pattern of a cloud of dots was towards the left or right of the screen. Each block contained trials that differed in the proportion of dots moving coherently in the correct direction, and blocks differed in either the instructions that participants received, the proportion of trials that had dots moving in each direction, or any combination of these effects. The proportion of dots moving coherently in the correct direction attempted to make some trials more difficult than others, being used as a direct manipulation of drift rate. The instructions that participants received differed between blocks in whether participants were encouraged to emphasize the accuracy of their performance, or the speed of their performance, being used as a direct manipulation of threshold (though see Voss et al., 2004 and Rae et al., 2014 for how instructions may influence other parameters). The proportion of trials that had dots moving in a specific direction was intended to create a response bias in participants, and used as a direction manipulation of bias.

Although the study of Dutilh et al., (2018) was collected as a single experiment, it was split into 14 “pseudo-experiments” that each had two conditions, which either differed in difficulty, instructions, bias, or any combination of these three effects. For the purpose of my assessment, I only use the five pseudo-experiments that fit within the framework of my previous simulations: a “none” experiment, a “drift” experiment, a “threshold” experiment, a “drift and threshold extreme” experiment, and a “drift and threshold balanced” experiment. In each experiment, I treat each participant as a replicate of that effect, and performed the same assessments as in the simulation study. In general, the size of the effect in response time for the drift rate manipulation was quite small (mean over participants = 0.248; SD = 0.198), whereas the size of the effect for the threshold manipulation was quite large (mean over participants = 0.964, SD = 0.423).

### Results

#### Selection of correct model

**Fig. 11:**
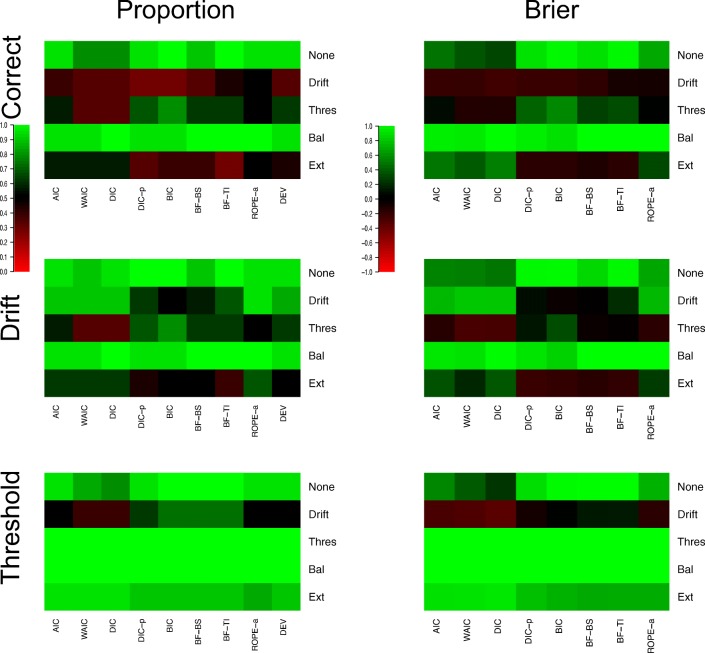
Plots of the proportion (*left panels*) and Brier scores (*right panels*) of correct selections (*top panels*), drift rate selections (*middle panels*), and threshold selections (*bottom panels*) for each model selection method (columns) for the five different cells of the design (rows). *Lighter shades of green* indicate more selections, *lighter shades of red* indicate less selections, and *black* indicates intermediate performance, which can be seen in the color bar to the left-hand side

To begin, I assessed how often each model selection method selected the “correct” model, based upon the selective influence assumption for each manipulation. The top-left panel of Fig. 11 displays the correct selections for each model selection method in each of the five cells of the pseudo-experimental design, and top-right panel displays the average Brier scores.

Overall, the performance of the methods is much worse here than within the simulated data, which could potentially be attributed to the selective influence assumption being incorrect (Voss et al., 2004; Rae et al., 2014), or the greater difficulty in making inferences within empirical data, where the model fit is unlikely to be the true generating process. Although performance is near-optimal in the “balanced” case and the no effect case, performance is worse in the other three classes of effects. AIC, DIC, and WAIC all perform poorly in identifying drift only and threshold only effects and decently in the extreme case, whereas DIC_*p*_, BIC, BF-BS, and BF-TI perform poorly in the drift and extreme cases, but perform well in the threshold case. ROPE_*a*_ appears to perform decently in all three of these classes of effects, suggesting that it may be performing the best of all of the methods.

In hindsight, the poor performance in the “extreme” data set and near-optimal performance in the “balanced” data set may be explained by the findings of Rae et al., (2014). Specifically, Rae et al., found that when given speed instructions, participants had both a lower threshold and a lower drift rate, suggesting that instructions also influence drift rate.^5^ Interestingly, in the “balanced” data these two drift rate effects move in the same direction, but in the “extreme” data they move in opposite directions. Therefore, the poor performance in the extreme data set could be directly attributed to a failure of the selective influence assumption, where there are opposing drift rate effects that balance one another.

#### Selection of specific effects

The reasons why each method performed poorly in certain cells are made clearer in the middle and bottom panels of Fig. 11, which display the proportion of drift and threshold selections, respectively. In the “balanced” and no effect cases, all models almost always select both effects and neither effect respectively, which follows from their near-optimal performance in these cells. Within the drift data, where all methods performed poorly, AIC, DIC, WAIC, and ROPE_*a*_ all select a drift rate effect in most data sets, whereas DIC_*p*_, BIC, BF-BS, and BF-TI select a drift rate effect a little over half the time. However, AIC, DIC, WAIC, and ROPE_*a*_ all also regularly select a threshold effect, whereas DIC_*p*_, BIC, BF-BS, and BF-TI rarely do. Therefore, although all methods perform poorly in these data, the reasons are quite different: AIC, DIC, WAIC, and ROPE_*a*_ all select an overly flexible model too often, and DIC_*p*_, BIC, BF-BS, and BF-TI all select an overly simple model too often, which is consistent with the findings of the simulated study. The other poor performances follow similar reasoning: AIC, DIC, WAIC, and ROPE_*a*_ often select both drift rate and threshold effects in the threshold data set, and DIC_*p*_, BIC, BF-BS, and BF-TI often select only the threshold effect in the “extreme” data set.

#### Consistency between metrics

**Fig. 12:**
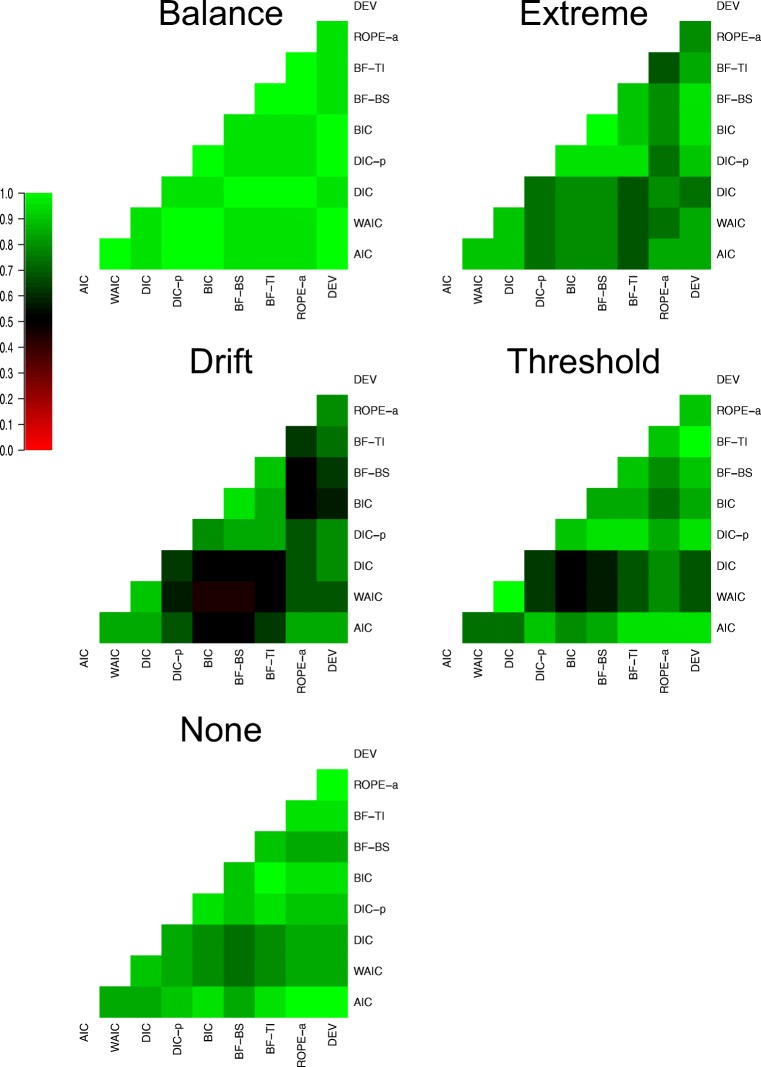
Plots the agreement in selected model between each of the eight model selection methods (rows and columns of each plot) for five cells of the design. *Lighter shades of green* indicate greater agreement, *lighter shades of red* indicate greater disagreement, and *black* indicating intermediate agreement, which can be seen in the color bar to the left-hand side

Figure 12 displays the proportion of data sets where the same model was selected by each of the different methods for each cell of the design. Interestingly, despite the “poor” performance of different methods in different cells of the design, the performance appears to be quite consistent between the methods overall. As expected from the analysis in the previous sub-sections, the agreement is highest in the “balanced” and no effect cells, with differences between methods being minimal. Differences appear to be largest within the single-effect data sets, and again appear to be clustered into groups, where the “Bayes factor” methods show high levels of agreement with one another, but differ from the “predictive accuracy” methods, which also show high levels of agreement with one another. However, two major exceptions to this rule appear to exist. Firstly, as with the simulated study, DIC_*p*_ is most consistent with the Bayes factor methods, and not very consistent with the standard DIC calculation. Secondly, in the threshold data, AIC shows closer agreement with the Bayes factor methods than the predictive accuracy methods. This could be due to either (1) a failing of the simple parameter counting approximation of AIC, or (2) the limited number of replicates with these data (20). However, in an overall sense, the simple approximation of AIC and BIC again show close agreement with the more complex methods that they aim to approximate, suggesting their adequacy in these types of comparisons between models.

## Discussion

The current study provided a systematic assessment of how different model selection methods perform in practical situations for the analysis of response time data using evidence accumulation models (EAMs). Specifically, I used the Linear Ballistic Accumulator (LBA; Brown and Heathcote, 2008), a simple and commonly applied model of decision-making, and compared two different types of theoretical accounts that are commonly of interest for researchers who apply these models: drift rate and decision threshold. My study compared 9 different model selection methods, including two general classes of model selection methods—predictive accuracy measures and Bayes factor approximations—which varied in their theoretical underpinnings, sophistication in dealing with model flexibility, and computational tractability. Although previous literature has shown theoretical differences between many of these methods, my study aimed to assess whether these differences would influence the inferences made in practical designs that EAMs are commonly applied to. I performed these comparisons for a large-scale simulation study, as well as the empirical data from Dutilh et al., (2018).

The findings of my study revealed two general patterns in the relationship between the different methods. Firstly, there were several large inconsistencies between the “predictive accuracy” methods (AIC, DIC, WAIC) and the “Bayes factor” methods (BIC, BF-BS, BF-TI) in the models selected, with the predictive accuracy methods generally being more liberal, and the Bayes factor methods generally being more conservative. Theoretically this result is not particularly surprising, as predictive accuracy methods will favor the more flexible model as the amount of data approaches infinite, and Bayes factors with uniformed priors are considered to be conservative. However, in practice these theoretical differences are often ignored, with a convenient method being applied as a general “model selection metric”, with the class of model selection method chosen rarely justified. These findings suggest that the theoretical difference is quite meaningful, and can often make a meaningful difference to the inferences about the data. In addition, one potentially interesting sub-finding is that these inconsistencies are only present when the effect of interest (e.g., drift rate) is non-existent or small, and that methods show extremely high consistency when the effect is moderate or large. Therefore, although there are inconsistencies between the classes of methods when any potential effects are small, when all potential effects are large then all methods provide equivalent results.

Secondly, there appeared to be few differences between the simple “parameter counting” methods of model selection, and the more complex variants that accounted for functional form flexibility. Specifically, within each class of methods discussed above, the simple approximations showed high levels of consistency with the more complex methods. AIC showed a high level of agreement with both DIC and WAIC, and BIC showed a high level of agreement with both Bayes factor approximation methods. Although it is known that the flexibility of the model is more than simply the sum of its free parameters, with the way that they are functionally combined also impacting upon the flexibility of the model, my findings appear to suggest that the parameter counting heuristic provides a fairly accurate approximation of flexibility when applying the LBA. These two general findings both appeared to be robust across both simulated and empirical data, suggesting that these findings should extrapolate to similar experimental context that the LBA might be applied to.

The findings of my study also revealed some more specific patterns in the relationship between the methods. Firstly, DIC and WAIC came to the same conclusions in almost every circumstance, suggesting that these methods are, for practical purposes, almost identical. Although WAIC has been motivated for its theoretical benefits over DIC, these do not appear to be practically applicable in the situations assessed here, suggesting WAIC provides little additional benefit over DIC when applying the LBA. Secondly, the addition of the prior probability to the DIC calculation (e.g., basing the deviance on *p*(*y*|***𝜃***)*p*(***𝜃***) instead of *p*(*y*|***𝜃***)) greatly changed its inferences, with DIC_*p*_ showing a closer relationship to the Bayes factor than to the standard DIC calculation. This suggests that when a researcher wants a measure of predictive accuracy, they should use the standard DIC calculation; however, if they are looking for a simple Bayesian method that provides a similar result to the Bayes factor, then DIC_*p*_ may be an interesting alternative. Thirdly, the augmentation of the estimation method ROPE appeared to perform quite well, and showed similar performance in many instances to the Bayes factor. Therefore, when the “zero region” for ROPE is chosen in a principled manner, the method appears to provide a promising method of practically comparing models. Fourthly, although bridge sampling and thermodynamic integration both provide an unbiased approximation of the marginal likelihood that has been shown to be accurate for simpler models where the marginal likelihood is solvable, there were some minor inconsistencies in their selections. This could potentially be due to either one or both methods having too greater approximation error in the limited number of samples that were used, or the fact that the TIDE method is a further approximation of the TI method (Evans and Annis, 2019) which may have given it greater approximation error. Future research should investigate the practical differences between these approximation methods across different models and numbers of samples. Lastly, the performance of the methods differed greatly across differing numbers of trials, with methods being poorer (overly conservative) when the number of trials were small. Interestingly, the performance of the Bayes factor methods remained similar to one another across all numbers of trials, whereas AIC and DIC deviated from one another by increasing amounts with increasing numbers of trials, where DIC provided a greater number of false alarms than AIC.

### Which model selection methods should researchers use?

In general, the most commonly used model selection methods can differ from one another in two key ways: their level of sophistication in dealing with flexibility, and their theoretical underpinning. However, choosing a specific method can be a difficult process due to the vast number of model selection methods available. Here, I attempt to make some useful recommendations for researchers who wish to apply the LBA—or other EAMs—to empirical data.

Firstly, the results of this study appear to indicate that the level of sophistication in dealing with flexibility has little consequence on the inferences, and therefore, researchers should feel comfortable choosing a simple method that they can efficiently and robustly implement, such as AIC or BIC. Although several papers have shown more sophisticated methods of model selection to be theoretically better than simpler methods, there appear to be few practical differences between them when applying the LBA. This logic also extends to DIC and WAIC, where there appear to be even fewer practical differences, meaning that if the goal of researchers is predictive accuracy, they would likely be best served applying whichever method comes default within their estimation package of choice. However, these consistencies do have two key exceptions: one for the predictive accuracy methods, and one for the Bayes factor methods. For predictive accuracy methods, the parameter counting AIC and the more sophisticated DIC begin to differ when the number of trials increases, with DIC appearing to more closely reflect what would be expected by leave-on-out cross validation than AIC. However, from an “inversion” standpoint, this actually results in DIC providing a worse performance than AIC. For Bayes factor methods, BIC only provides a close approximation to the Bayes factor with uninformative priors on the parameters, and when informative priors are implemented on the parameter value difference between conditions, the Bayes factor becomes much less conservative, and improves from an “inversion” standpoint. Therefore, in cases where researchers wish to use informed priors, more sophisticated methods of marginal likelihood approximation should be used over BIC.

Secondly, the results of this study appear to indicate that the class of model selection method used—predictive accuracy or Bayes factor—can have major consequences for the inferences made. Therefore, researchers should carefully consider and justify the class of model selection method that they choose to implement (i.e., not just blindly applying a random class of method). Although both classes of methods show near-perfect agreement when a moderate or large effect is present, with both always selecting in favor of the effect, the classes diverge in the cases of small or null effects. However, the choice between the classes of methods may be difficult for many users of the LBA, as they may not have a theoretical preference for either “predictive accuracy” or “the best account of these data”, and the Bayes factor can also be thought of in terms of a different type of “predictive accuracy” (Rouder & Morey, 2018), meaning that the choice may seem arbitrary. In these situations, I would recommend choosing a class of method based on the treatment of model flexibility, or the practical impacts observed in this study. In terms of choices based on the treatment of model flexibility, predictive accuracy methods can be viewed as treating flexibility as the ability of models to overfit to the noise in a sample of data, as they only penalize models based on the observed flexibility that results in this overfitting. In contrast, Bayes factor methods can be viewed as treating flexibility as the ability of models to predict many different patterns of data, as they penalize models for having a highly spread prior probability. Therefore, researchers who believe that models should only be penalized based on overfitting to noise in data should use predictive accuracy methods, and researchers who believe that models should also be penalized for having a large range of a-priori predictions should use Bayes factor methods. In terms of choices based on the practical impacts, this study showed that predictive accuracy methods are generally more liberal, detecting most effects that are present while also providing some false alarms, whereas Bayes factor methods are generally more conservative, rarely providing false alarms while also missing many small effects. Therefore, researchers who are more worried about missing effects should use predictive accuracy methods, and researchers who are more worried about incorrectly detecting effects should use Bayes factor methods.

Lastly, the results of this study appear to indicate that considering the probability assigned to each model results in better performance (by inversion standards). Therefore, it is important that researchers consider both which model is chosen as the best model by the model selection method, as well as the strength of evidence in favor of the winning model over the other models. Previous applications of the LBA have largely focused on only which model was selected as the best model, and have mostly ignored the strength of evidence in the selection. However, this study found that performance was improved for all model selection methods when accounting for the probability assigned to each model, suggesting that the methods are more likely to show high confidence in selecting correct models than incorrect models. The strength of evidence in favor of each model can be easily obtained for all of the methods used within this manuscript (excluding the *χ*^2^ test of relative deviance) by expressing the selection metric as relative probability weights for each model (e.g., Hawkins, Forstmann, Wagenmakers, Ratcliff, & Brown, 2015).

### How should informed priors be selected for Bayes factors?

As discussed above, BIC provides similar performance to Bayes factors with uninformed priors when testing psychological theories within the LBA framework, making advanced marginal likelihood estimation methods of limited value in these situations. However, Bayes factors become less conservative when more informed priors are used, meaning that researchers may wish to apply Bayes factors with informed priors on the effect parameters. The choice of priors is largely subjective, and can be highly influential in the resulting model chosen, meaning that informed priors must be chosen with care. Although recommending exact informed priors for researchers to use is beyond the scope of this study, in this section I attempt to provide a brief, general discussion of some of the different ways for *how* informed priors might be developed. Specifically, I discuss three broad potential approaches for conceptualizing and developing informed priors: priors as a belief, priors as previous information, and priors as an optimal inference tool. Readers who are interested in developing or using informed priors should also see Lee and Vanpaemel (2018), who provide a much more comprehensive discussion of informed priors for cognitive models.

#### Priors as a belief

Within the Bayesian framework, priors are often posed as the subjective belief that a person has about probability of each possible outcome before having observed the current data (Jeffreys & Wrinch, 1921). Under this interpretation the posterior is the subjective belief for each possible outcome after having observed the current data, with the Bayes factor being the change in belief from the prior to the posterior based on the current data. Subjective beliefs provide a convenient method for creating informed priors, as the prior is simply whatever the researcher believes a-priori. For example, in the case of the LBA, a researcher might believe that the difference in drift rate between experimental conditions based on a randomly chosen manipulation is most likely to be 0, that positive and negative effects are equally likely, and that smaller effects are more likely than larger effects, with differences above 3 in magnitude being highly unlikely, but still possible. Based on this verbal description of the researcher’s belief, a normal distribution (i.e., smaller effects are more likely than larger effects) with a mean of 0 (i.e., most likely value of 0) and standard deviation of 1 (i.e., values with magnitude above 3 occur in approximately 0.25% of cases) might provide a practical formalized representation of their belief that can be used as the prior distribution.

However, some researchers view the subjective nature of belief-based priors as being problematic, as researchers with different a priori subjective beliefs may make inconsistent inferences about the data (see Vanpaemel, 2010; Lee & Vanpaemel, 2018 for discussions). Using the above example, a different researcher may believe that differences above 0.3 in magnitude are highly unlikely, resulting in their use of a much tighter prior with a standard deviation of 0.1. As shown within this study, this exact difference in prior will often result in different inferences being made, meaning that the researchers could make different conclusions about whether a drift rate effect is present or not within the same data set. One option to try and make “priors as a belief” more objective is *expert elicitation* (Kadane & Wolfson, 1998; Gronau, Ly, & Wagenmakers, 2017). Specifically, expert elicitation involves attempting to define a prior based on the overall belief of experts within the field, where information about what the distribution should look like is elicited from these experts through a series of questions. Importantly, expert elicitation provides a prior that is a subjective belief, but also allows the prior to be “objective” in the sense of being consistent across researchers. Indeed, expert elicitation has been used in simpler statistical models to obtain informed priors for Bayes factors (Gronau et al., 2017), and provides a promising, and potentially easy to obtain, method of forming agreed-upon informed priors for cognitive models.

#### Priors as previous information

One of the key benefits of the Bayesian framework is the ability to easily provide cumulative knowledge. As in the famous adage “today’s posterior is tomorrow’s prior” (Lindley, 1972; Wagenmakers, Lodewyckx, Kuriyal, & Grasman, 2010), the posterior distributions from one study can be used as the prior distributions for subsequent studies, such as extensions or replications. This process can allow information from previous studies to be directly integrated into future studies, allowing precise estimates for parameter values to be developed over numerous experiments. However, these ideals can also be applied more generally to develop informed priors for the parameters of models (see Lee & Vanpaemel, 2018 for a detailed discussion), rather than precise posterior distributions for only a single experimental paradigm. For example, in the case of the LBA, a researcher could obtain the data from a large number of empirical studies and estimate the difference in drift rates between all of the experimental manipulations in all of the studies. These estimates could then be used to develop an informed prior that is reflective of what drift rate differences have been observed in previous studies. Importantly, this approach allows the removal of some of the subjective nature of priors, with the informed priors being formed based on empirical observations and developed according to clear, objective principles.

Although developing informed priors from previous experimental data provides a promising and objective method of developing informed priors, the approach also contains some potential limitations. Firstly, the approach of using previous posterior distributions for future priors makes the strong assumption that the same parameter is being estimated in both situations. However, this is not as simple as using the same model and parameterization: changes in experimental context may result in fundamental changes to the underlying parameter value, meaning that the previous posterior would no longer be an appropriate prior for the new context. Therefore, care has to be taken to ensure that informed priors developed through this method are not extrapolated beyond the contexts that informed them. Secondly, recent research has suggested that empirically based priors are of limited meaning and value in unidentifiable models (Spektor & Kellen, 2018). Importantly, EAMs such as the LBA are known to be “sloppy” models, which have degeneracy in the parameter values due to high correlations between the parameters (Holmes, 2015), meaning that empirical based priors may not be the best option for the LBA.

#### Priors as an optimal inference tool

Another potential way that priors could be thought about are as tools for inference. Rather than a prior being a belief, or the results from previous data, the Bayesian framework could potentially incorporate priors that attempt to optimize the detection of effects, as the findings of this study suggested that the detection properties differ greatly based on the priors. Although priors are commonly based on “belief” or “information” (though see Rouder et al., 2012 for a discussion of how default priors can be useful), my reason for suggesting priors as tools for inference has a similar underpinning to that of optimal experimental designs (e.g., Myung & Pitt, 2009). Specifically, optimal experimental designs attempt to maximize the amount of information obtained in an experiment – or minimize the time the participants have to spend on an experiment—by incorporating the information already obtained and using it to provide participants with trials that maximize information gain. Recent research has also developed optimal design analysis in the Bayesian framework for simple statistical models (Schönbrodt & Wagenmakers, 2018; Stefan, Gronau, Schönbrodt, & Wagenmakers, 2018), which assesses how likely an experimental design is to provide strong evidence for a hypothesis, allowing researchers to choose their sample size, or in the case of Bayesian optional stopping the evidence boundaries, to maximize information gain. Perhaps a similar logic could be applied with priors, allowing researchers to see how different choices of priors would effect their detection of effects within their proposed experimental design, and allowing them to choose a prior—before analyzing the data—that provides optimal properties. Future research may benefit from a detailed exploration of how priors can be selected in a manner that can optimize the statistical properties of the Bayes factor.

### What about hierarchical models?

Hierarchical models have become a popular method over the last decade of estimating the parameters of cognitive models (Shiffrin, Lee, Kim, & Wagenmakers, 2008; see Evans & Brown, 2017 and Evans, Brown, Mewhort, & Heathcote, 2018 for applications). Although a “hierarchical model” can technically refer to any model with some parameters that are structured hierarchically, hierarchical cognitive models most commonly involve Bayesian parameter estimation on groups of participants, where the parameters of the model are estimated for each participant, and the parameters of these participants are constrained to follow some group level distribution. For example, in a hierarchical LBA, the correct drift rate of a null model is commonly defined as:

$$\begin{array}{@{}rcl@{}} v_{c,s} & \sim & TN(\mu_{v_{c}},\sigma_{v_{c}},0,Inf) \end{array} $$

where *s* indexes participants. In contrast, a model with an difference in correct drift rate between conditions is commonly defined as:

$$\begin{array}{@{}rcl@{}} v_{c,i,s} & \sim & TN(\mu_{v_{c,i}},\sigma_{v_{c,i}},0,Inf) \end{array} $$

*i* indexes the condition.

Despite the increasing popularity of hierarchical models, I decided to exclude them from my assessment for two main reasons. Firstly, the inclusion of multiple participants per data set in the simulation study would have increased the computational burden drastically (i.e., by a factor of the number of participants included), meaning that the assessment likely would have had to be less comprehensive to fit into a reasonable computational time frame. Therefore, I chose to perform a more comprehensive and robust assessment on non-hierarchical models, rather than a lesser version involving hierarchical models.

Secondly, and most importantly, there are many potential theoretical difficulties when performing comparisons between nested hierarchical models (i.e., using them as “measurement tools”), which are still largely unresolved within the literature. Previous research has already highlighted the issues with “two-stage” approaches, where model parameters are estimated with hierarchical cognitive models and then placed into a subsequent statistical test (e.g., a t-test), as this will lead to biases in favor of an effect (Boehm, Marsman, Matzke, & Wagenmakers, 2018). However, the potential issues are not limited to estimation-based approaches to hierarchical model comparisons. Specifically, there are three key statistically relevant ways that the hierarchical models can potentially differ from one another: whether or not they allow (1) each participant to have a drift rate value that differs from other participants (i.e., a “random intercept”; see Singmann & Kellen, 2018 for explanations of different types of “random effects”), (2) each participant to have a difference between their drift rate values that differs from other participants (i.e., a “random slope”), and (3) the average drift rate value to differ from some fixed value (i.e., 0; the “fixed effect” of interest). In the hierarchical model definitions above, the common “null” hierarchical model only allowed random intercepts, constraining all participant slopes to take on the same, fixed value of 0. In contrast, the common “drift” hierarchical model allows all three of these freedoms.

Although this second reason for not assessing hierarchical models may seem like a niche statistical point, not properly considering this issue can have meaningful theoretical consequences on the inferences made. In the example above, the null model makes the very strong assumption that there are no individual differences between participants in their difference in drift rate between conditions, due to not having random slopes: essentially, the model allows no random variability in the effect. Therefore, even if the true drift rate effect is centered on 0 (i.e., no effect), having some amount of between-person variability in the exact drift rate difference between conditions will result in the “drift” model being the superior model. However, it is not clear whether this strong assumption is a reasonable one to make, and researchers may differ on their opinions of whether or not the null model should include random slopes. In addition, if random slopes are deemed to be necessary for null models, then assessments still need to be made on the best way to define this new hierarchical null model. The “random slopes” issue is also not restricted to comparisons made using Bayesian hierarchical model selection; the same logic and strong constraints (i.e., no random variability) applies to group-based comparisons made using non-hierarchical models, such as summing the AIC or BIC values over participants (e.g., Rae et al., 2014). Therefore, although I believe the assessment of whether the findings of this article generalize to hierarchical models is important, I also believe that this is best left for future research when the random slopes issue in hierarchical cognitive models has been resolved.

### Limitations and future research

Although I believe the current study has many useful findings, as highlighted above, there are also several limitations that should be acknowledged. First and foremost, my study only focused on how these methods relate to one another for applications of the LBA. Although it seems plausible that these findings would transfer to similar EAMs, such as the diffusion model, this is not necessarily the case, and future research should explore whether the general patterns of results hold for other models. Secondly, in order to minimize the potential sources of variability in evaluating the consistency of the methods in my simulated study, I used a fairly restricted range of parameter values in generating the data, with these values being based on my past experiences of fitting the LBA to empirical data. Although this limitation was somewhat resolved by also assessing empirical data, future assessments on simulated data could potentially focus on specific cells of my design that I found to cause the greatest inconsistencies between methods, and explore a greater portion of the parameter space within them. Lastly, it should be noted that my assessment only covered within-model comparisons, using the LBA as a nested measurement tool. In the context of these within-model comparisons, the functional form differences between the theoretical accounts is limited, which might explain why accounting for functional form flexibility did not provide much of a difference to simple parameter counting. However, when comparing between different classes of models to compare how well they explain the decision-making process, the functional form may differ a great deal between the models, and accounting for functional form flexibility might become more important. Therefore, the results of this study should probably be restricted to applications of EAMs as measurement tools.

## Electronic supplementary material

Below is the link to the electronic supplementary material.

(PDF 128 KB)

## Footnotes

1 Note that Osth et al., (2017) used a hierarchical model and only included part of the prior—the probability of the individual level parameters given the hyper-parameters [i.e., *p*(***𝜃***|***ϕ***)]—in the calculation, meaning that they did not use the full unnormalized posterior probability as I have here.

2 It should be noted that the *extreme* and *balancing* effects are within the response times, and that these parameter combinations actually have the opposite effect on accuracy. However, this is not meant to imply that response time is more important than accuracy, and the “extreme” and “balance” naming is only used for clarity.

3 Note that for every figure, an equivalent table with the exact numerical values can be found in the Appendix.

4 Note that I use the *difference* between conditions instead of the *effect size* used in statistical models, as there is no standard deviation in the parameter estimates to normalize the difference by.

5 However, note that because of the scaling degeneracy in EAMs, this lowering of both parameters may have actually reflected an increase in the accumulation noise.

## References

[CR1] Akaike H (1974). A new look at the statistical model identification. IEEE Transactions on Automatic Control.

[CR2] Annis, J., Evans, N.J., Miller, B.J., & Palmeri, T.J. (2018). Thermodynamic integration and steppingstone sampling methods for estimating Bayes factors: A tutorial. Retrieved from https://psyarxiv.com/r8sgn 10.1016/j.jmp.2019.01.005 PMC6374050 30774151

[CR3] Boehm, U., Marsman, M., Matzke, D., & Wagenmakers, E.-J. (2018). On the importance of avoiding shortcuts in applying cognitive models to hierarchical data. *Behavior Research Methods*, 1–18. 10.3758/s13428-018-1054-3 PMC6096647 29949071

[CR4] Box GE, Draper NR (1987). Empirical model-building and response surfaces.

[CR5] Brier GW (1950). Verification of forecasts expressed in terms of probability. Monthey Weather Review.

[CR6] Brown SD, Heathcote A (2008). The simplest complete model of choice response time: Linear ballistic accumulation. Cognitive Psychology.

[CR7] Brown SD, Marley AAJ, Donkin C, Heathcote A (2008). An integrated model of choices and response times in absolute identification. Psychological Review.

[CR8] Donkin C, Averell L, Brown S, Heathcote A (2009). Getting more from accuracy and response time data: Methods for fitting the linear ballistic accumulator. Behavior Research Methods.

[CR9] Donkin C, Brown SD, Heathcote A (2009). The overconstraint of response time models: Rethinking the scaling problem. Psychonomic Bulletin and Review.

[CR10] Dutilh, G., Annis, J., Brown, S.D., Cassey, P., Evans, N.J., Grasman, R.P., & et al. (2018). The quality of response time data inference: A blinded, collaborative assessment of the validity of cognitive models. *Psychonomic bulletin and review*, 1–19. 10.3758/s13423-017-1417-2 PMC6449220 29450793

[CR11] Dutilh G, Vandekerckhove J, Tuerlinckx F, Wagenmakers E-J (2009). A diffusion model decomposition of the practice effect. Psychonomic Bulletin and Review.

[CR12] Evans, N.J., & Annis, J. (2019). Thermodynamic integration via differential evolution: A method for estimating marginal likelihoods. *Behavior Research Methods*, 1–18. 10.3758/s13428-018-1172-y PMC6478771 30604038

[CR13] Evans, N.J., Bennett, A.J., & Brown, S.D. (2018). Optimal or not; depends on the task. *Psychonomic Bulletin and Review*, 1–8. 10.3758/s13423-018-1536-4 PMC6557863 30411197

[CR14] Evans NJ, Brown SD (2017). People adopt optimal policies in simple decision-making, after practice and guidance. Psychonomic Bulletin and Review.

[CR15] Evans NJ, Brown SD (2018). Bayes factors for the linear ballistic accumulator model of decision-making. Behavior Research Methods.

[CR16] Evans NJ, Brown SD, Mewhort DJ, Heathcote A (2018). Refining the law of practice. Psychological Review.

[CR17] Evans NJ, Hawkins GE, Boehm U, Wagenmakers E-J, Brown SD (2017). The computations that support simple decision-making: A comparison between the diffusion and urgency-gating models. Scientific Reports.

[CR18] Evans NJ, Howard ZL, Heathcote A, Brown SD (2017). Model flexibility analysis does not measure the persuasiveness of a fit. Psychological Review.

[CR19] Evans, N.J., Rae, B., Bushmakin, M., Rubin, M., & Brown, S.D. (2017). Need for closure is associated with urgency in perceptual decision-making. *Memory and Cognition*, 1–13. 10.3758/s13421-017-0718-z 28585159

[CR20] Evans NJ, Steyvers M, Brown SD (2018). Modeling the covariance structure of complex datasets using cognitive models: An application to individual differences and the heritability of cognitive ability. Cognitive Science.

[CR21] Forstmann BU, Dutilh G, Brown S, Neumann J, Von Cramon DY, Ridderinkhof KR, Wagenmakers E-J (2008). Striatum and pre-SMA facilitate decision-making under time pressure. Proceedings of the National Academy of Sciences.

[CR22] Forstmann BU, Tittgemeyer M, Wagenmakers E-J, Derrfuss J, Imperati D, Brown S (2011). The speed–accuracy tradeoff in the elderly brain: A structural model-based approach. The Journal of Neuroscience.

[CR23] Friel N, Hurn M, Wyse J (2014). Improving power posterior estimation of statistical evidence. Statistics and Computing.

[CR24] Friel N, Wyse J (2012). Estimating the evidence–a review. Statistica Neerlandica.

[CR25] Grasman RP, Wagenmakers E-J, Van Der Maas HL (2009). On the mean and variance of response times under the diffusion model with an application to parameter estimation. Journal of Mathematical Psychology.

[CR26] Gronau, Q.F., Ly, A., & Wagenmakers, E.-J. (2017). Informed Bayesian *t*-tests. arXiv:1704.02479

[CR27] Gronau QF, Sarafoglou A, Matzke D, Ly A, Boehm U, Marsman M, Steingroever H, ... (2017). A tutorial on bridge sampling. Journal of Mathematical Psychology.

[CR28] Gronau, Q.F., & Wagenmakers, E.-J. (2018). Limitations of Bayesian leave-one-out cross-validation for model selection. *Computational Brain and Behavior*, 1–11. 10.1007/s42113-018-0011-7 PMC6400414 30906917

[CR29] Hawkins GE, Forstmann BU, Wagenmakers E-J, Ratcliff R, Brown SD (2015). Revisiting the evidence for collapsing boundaries and urgency signals in perceptual decision-making. The Journal of Neuroscience.

[CR30] Hawkins GE, Marley A, Heathcote A, Flynn TN, Louviere JJ, Brown SD (2014). The best of times and the worst of times are interchangeable. Decision.

[CR31] Hawkins GE, Marley A, Heathcote A, Flynn TN, Louviere JJ, Brown SD (2014). Integrating cognitive process and descriptive models of attitudes and preferences. Cognitive Science.

[CR32] Ho TC, Yang G, Wu J, Cassey P, Brown SD, Hoang N (2014). Functional connectivity of negative emotional processing in adolescent depression. Journal of Affective Disorders.

[CR33] Holmes WR (2015). A practical guide to the probability density approximation (PDA) with improved implementation and error characterization. Journal of Mathematical Psychology.

[CR34] Jeffreys H, Wrinch D (1921). On certain fundamental principles of scientific enquiry. Philosophical Magazine.

[CR35] Kadane J, Wolfson LJ (1998). Experiences in elicitation. Journal of the Royal Statistical Society: Series D (The Statistician).

[CR36] Kass RE, Raftery AE (1995). Bayes factors. Journal of the American Statistical Association.

[CR37] Kruschke JK (2011). Bayesian assessment of null values via parameter estimation and model comparison. Perspectives on Psychological Science.

[CR38] Kruschke JK, Liddell TM (2018). The bayesian new statistics: Hypothesis testing, estimation, meta-analysis, and power analysis from a Bayesian perspective. Psychonomic Bulletin and Review.

[CR39] Lee, M.D. (2018). Bayesian methods in cognitive modeling. *Stevens handbook of experimental psychology and cognitive neuroscience*, 37–84.

[CR40] Lee MD, Vanpaemel W (2018). Determining informative priors for cognitive models. Psychonomic Bulletin and Review.

[CR41] Lindley DV (1957). A statistical paradox. Biometrika.

[CR42] Lindley DV (1972). Bayesian statistics, a review, Vol. 2.

[CR43] Liu P, Elshall AS, Ye M, Beerli P, Zeng X, Lu D, Tao Y (2016). Evaluating marginal likelihood with thermodynamic integration method and comparison with several other numerical methods. Water Resources Research.

[CR44] Matzke D, Dolan CV, Logan GD, Brown SD, Wagenmakers E-J (2013). Bayesian parametric estimation of stop-signal reaction time distributions. Journal of Experimental Psychology: General.

[CR45] Meng, X.-L., & Wong, W.H. (1996). Simulating ratios of normalizing constants via a simple identity: A theoretical exploration. *Statistica Sinica*, 831–860.

[CR46] Myung IJ (2000). The importance of complexity in model selection. Journal of Mathematical Psychology.

[CR47] Myung IJ, Pitt MA (1997). Applying Occam’s razor in modeling cognition: A Bayesian approach. Psychonomic Bulletin and Review.

[CR48] Myung IJ, Pitt MA (2009). Optimal experimental design for model discrimination. Psychological Review.

[CR49] Osth AF, Dennis S, Heathcote A (2017). Likelihood ratio sequential sampling models of recognition memory. Cognitive Psychology.

[CR50] Rae B, Heathcote A, Donkin C, Averell L, Brown S (2014). The hare and the tortoise: Emphasizing speed can change the evidence used to make decisions. Journal of Experimental Psychology: Learning Memory, and Cognition.

[CR51] Ratcliff R (1978). A theory of memory retrieval. Psychological Review.

[CR52] Ratcliff R, Smith PL, Brown SD, McKoon G (2016). Diffusion decision model: Current issues and history. Trends in Cognitive Sciences.

[CR53] Ratcliff R, Thapar A, McKoon G (2001). The effects of aging on reaction time in a signal detection task. Psychology and Aging.

[CR54] Roberts S, Pashler H (2000). How persuasive is a good fit? a comment on theory testing. Psychological Review.

[CR55] Rouder, J.N., & Morey, R.D. (2018). Teaching Bayes theorem: Strength of evidence as predictive accuracy. *The American Statistician*, 1–5.

[CR56] Rouder JN, Morey RD, Speckman PL, Province JM (2012). Default Bayes factors for ANOVA designs. Journal of Mathematical Psychology.

[CR57] Salthouse TA (1996). The processing-speed theory of adult age differences in cognition. Psychological Review.

[CR58] Schönbrodt FD, Wagenmakers E-J (2018). Bayes factor design analysis: Planning for compelling evidence. Psychonomic Bulletin and Review.

[CR59] Schwarz G (1978). Estimating the dimension of a model. The Annals of Statistics.

[CR60] Shiffrin RM, Lee MD, Kim W, Wagenmakers E-J (2008). A survey of model evaluation approaches with a tutorial on hierarchical Bayesian methods. Cognitive Science.

[CR61] Singmann, H., & Kellen, D. (2018). An introduction to mixed models for experimental psychology. In D.H. Spieler, & E. Schumacher (Eds.) *New methods in neuroscience and cognitive psychology*: Psychology Press.

[CR62] Spektor, M.S., & Kellen, D. (2018). The relative merit of empirical priors in non-identifiable and sloppy models: Applications to models of learning and decision-making. *Psychonomic bulletin and review*, 1–22. 10.3758/s13423-018-1446-5 29589289

[CR63] Spiegelhalter DJ, Best NG, Carlin BP, Van Der Linde A (2002). Bayesian measures of model complexity and fit. Journal of the Royal Statistical Society: Series B (Statistical Methodology).

[CR64] Starns JJ, Ratcliff R (2012). Age-related differences in diffusion model boundary optimality with both trial-limited and time-limited tasks. Psychonomic Bulletin and Review.

[CR65] Stefan, A., Gronau, Q.F., Schönbrodt, F., & Wagenmakers, E.-J (2018). A tutorial on Bayes factor design analysis with informed priors. 10.3758/s13428-018-01189-8 PMC6538819 30719688

[CR66] Stone M (1960). Models for choice-reaction time. Psychometrika.

[CR67] Ter Braak CJ (2006). A Markov chain Monte Carlo version of the genetic algorithm differential evolution: Easy Bayesian computing for real parameter spaces. Statistics and Computing.

[CR68] Tillman G, Benders T, Brown SD, van Ravenzwaaij D (2017). An evidence accumulation model of acoustic cue weighting in vowel perception. Journal of Phonetics.

[CR69] Trueblood JS, Brown SD, Heathcote A, Busemeyer JR (2013). Not just for consumers: Context effects are fundamental to decision making. Psychological Science.

[CR70] Turner BM, Forstmann BU, Wagenmakers E-J, Brown SD, Sederberg PB, Steyvers M (2013). A Bayesian framework for simultaneously modeling neural and behavioral data. NeuroImage.

[CR71] Turner BM, Sederberg PB, Brown SD, Steyvers M (2013). A method for efficiently sampling from distributions with correlated dimensions. Psychological Methods.

[CR72] Usher M, McClelland JL (2001). The time course of perceptual choice: The leaky, competing accumulator model. Psychological Review.

[CR73] van Ravenzwaaij D, Brown S, Wagenmakers E-J (2011). An integrated perspective on the relation between response speed and intelligence. Cognition.

[CR74] van Ravenzwaaij D, Dutilh G, Wagenmakers E-J (2012). A diffusion model decomposition of the effects of alcohol on perceptual decision making. Psychopharmacology.

[CR75] Vanpaemel W (2010). Prior sensitivity in theory testing: An apologia for the Bayes factor. Journal of Mathematical Psychology.

[CR76] Vehtari A, Gelman A, Gabry J (2017). Practical Bayesian model evaluation using leave-one out cross-validation and WAIC. Statistics and Computing.

[CR77] Voss A, Rothermund K, Voss J (2004). Interpreting the parameters of the diffusion model: An empirical validation. Memory and Cognition.

[CR78] Wagenmakers E-J (2007). A practical solution to the pervasive problems of p values. Psychonomic Bulletin and Review.

[CR79] Wagenmakers E-J, Lodewyckx T, Kuriyal H, Grasman R (2010). Bayesian hypothesis testing for psychologists: A tutorial on the Savage–Dickey method. Cognitive Psychology.

[CR80] Wagenmakers E-J, Van Der Maas HL, Grasman RP (2007). An EZ-diffusion model for response time and accuracy. Psychonomic Bulletin and Review.

[CR81] Xie W, Lewis PO, Fan Y, Kuo L, Chen M-H (2010). Improving marginal likelihood estimation for Bayesian phylogenetic model selection. Systematic Biology.

[CR82] Yarkoni T, Westfall J (2017). Choosing prediction over explanation in psychology: Lessons from machine learning. Perspectives on Psychological Science.

