# Supplementary material for: Assessing the practical differences between model selection methods in inferences about choice response time tasks
Source: Psychon Bull Rev. 2019 Feb 19;26(4):1070–98. doi: 10.3758/s13423-018-01563-9 (PMC6710222; doi:10.3758/s13423-018-01563-9)
Supplement: Supplementary file 1 — (PDF 128 KB) [file 13423_2018_1563_MOESM1_ESM.pdf]

Appendix

Exact values from all figures

The following tables provide the exact numerical values from each of the figures in the main text, as the figures in text only provide a broad, general patterns in the data. Specifically, there is a single table for each figure, presented in the same order.

Table A1: Corresponds to Figure 1. The first three columns refer to the drift effect (null, small, moderate, large), threshold effect (null, small moderate, large), and when both effects are present whether they are balanced or extreme. Numbers listed here are the number of correct model identifications out of a possible 100.

D	T	B/E	AIC	WAIC	DIC	DIC_p	BIC	BF-BS	BF-TI	$ROPE_a$	DEV
L	L	B	100	100	100	100	100	100	100	100	100
L	L	E	100	100	100	100	100	100	100	100	100
L	M	B	100	100	100	100	100	100	99	100	100
L	M	E	99	99	99	99	98	98	97	99	99
L	S	B	78	83	81	48	35	40	41	61	57
L	S	E	79	83	84	50	34	24	25	60	59
M	L	B	100	100	100	96	96	98	97	99	100
M	L	E	100	100	100	99	96	95	95	100	100
M	M	B	100	100	100	100	99	99	99	100	100
M	M	E	100	100	100	98	93	97	96	98	100
M	S	B	73	80	81	50	39	35	43	57	54
M	S	E	77	78	80	45	29	27	25	47	54
S	L	B	62	72	70	38	26	41	33	54	46
S	L	E	61	69	69	29	24	26	31	47	41
S	M	B	73	79	81	43	28	32	34	56	50
S	M	E	75	80	81	38	33	34	37	55	53
S	S	B	51	64	67	21	12	14	11	35	53
S	S	E	42	51	51	6	2	1	4	9	12
L	N		85	74	76	97	99	99	99	95	94
M	N		78	70	72	96	99	99	98	91	95
S	N		69	69	68	63	60	63	60	41	72
N	L		87	73	71	99	100	93	94	93	96
N	M		83	65	62	97	100	96	97	94	96
N	S		76	56	55	72	65	53	53	49	76
N	N		78	57	56	98	99	99	97	95	90

Table A2: Corresponds to Figure 2. The first three columns refer to the drift effect (null, small, moderate, large), threshold effect (null, small moderate, large), and when both effects are present whether they are balanced or extreme. Numbers listed here are the average Brier scores for model selections.

D	T	B/E	AIC	WAIC	DIC	DIC_p	BIC	BF-BS	BF-TI	$ROPE_a$
L	L	B	1	1	1	1	1	1	1	1
L	L	E	1	1	1	1	1	1	1	1
L	M	B	1	1	1	1	1	1	0.98	1
L	M	E	0.99	0.99	0.99	0.98	0.96	0.95	0.93	0.98
L	S	B	0.65	0.72	0.71	0.14	-0.14	-0.14	-0.12	0.32
L	S	E	0.65	0.71	0.72	0.15	-0.16	-0.28	-0.29	0.27
M	L	B	1	0.99	0.99	0.94	0.93	0.97	0.96	0.98
M	L	E	1	1	1	0.95	0.92	0.9	0.91	0.98
M	M	B	1	1	1	0.99	0.97	0.97	0.96	0.99
M	M	E	0.99	0.99	1	0.95	0.89	0.92	0.91	0.97
M	S	B	0.62	0.7	0.71	0.17	-0.13	-0.14	-0.09	0.23
M	S	E	0.62	0.66	0.66	0.03	-0.22	-0.29	-0.33	0.05
S	L	B	0.47	0.58	0.58	-0.11	-0.3	-0.08	-0.15	0.09
S	L	E	0.48	0.54	0.56	-0.19	-0.33	-0.24	-0.22	-0.01
S	M	B	0.59	0.66	0.68	-0.03	-0.24	-0.11	-0.11	0.19
S	M	E	0.6	0.68	0.7	-0.03	-0.19	-0.1	-0.07	0.12
S	S	B	0.3	0.44	0.46	-0.28	-0.53	-0.55	-0.54	-0.09
S	S	E	0.2	0.26	0.28	-0.42	-0.58	-0.54	-0.57	-0.34
L	N		0.58	0.45	0.45	0.89	0.97	0.97	0.94	0.85
M	N		0.55	0.42	0.42	0.88	0.96	0.97	0.92	0.81
S	N		0.39	0.33	0.34	0.37	0.27	0.33	0.28	-0.07
N	L		0.63	0.46	0.41	0.92	0.98	0.85	0.89	0.89
N	M		0.6	0.37	0.32	0.9	0.98	0.89	0.92	0.87
N	S		0.4	0.23	0.21	0.47	0.35	0.14	0.11	0.07
N	N		0.33	0.15	0.13	0.83	0.95	0.96	0.92	0.84

Table A3: Corresponds to Figure 3. The first three columns refer to the drift effect (null, small, moderate, large), threshold effect (null, small moderate, large), and when both effects are present whether they are balanced or extreme. Numbers listed here are the number of correct drift identifications out of a possible 100.

D	T	B/E	AIC	WAIC	DIC	DIC_p	BIC	BF-BS	BF-TI	$ROPE_a$	DEV
L	L	B	100	100	100	100	100	100	100	100	100
L	L	E	100	100	100	100	100	100	100	100	100
L	M	B	100	100	100	100	100	100	100	100	100
L	M	E	100	100	100	100	100	100	100	100	100
L	S	B	100	100	100	100	100	100	100	100	100
L	S	E	100	100	100	100	100	100	100	100	100
M	L	B	100	100	100	96	96	98	97	99	100
M	L	E	100	100	100	99	96	95	95	100	100
M	M	B	100	100	100	100	99	99	99	100	100
M	M	E	100	100	100	99	96	100	98	100	100
M	S	B	100	100	100	100	100	100	100	100	100
M	S	E	100	100	100	96	96	97	96	99	99
S	L	B	62	72	70	38	26	41	33	54	46
S	L	E	61	69	69	29	24	26	31	47	41
S	M	B	73	79	81	43	28	32	34	56	50
S	M	E	75	80	81	38	33	35	38	55	53
S	S	B	58	72	74	24	13	15	15	52	59
S	S	E	60	66	69	42	43	48	48	42	45
L	N		100	100	100	100	100	100	100	100	100
M	N		100	100	100	100	100	100	100	100	100
S	N		79	89	92	64	60	63	61	43	74
N	L		87	73	71	99	100	93	94	93	96
N	M		83	65	62	97	100	96	97	94	96
N	S		78	58	57	87	90	83	83	94	85
N	N		89	70	69	100	100	99	97	96	94

Table A4: Corresponds to Figure 4. The first three columns refer to the drift effect (null, small, moderate, large), threshold effect (null, small moderate, large), and when both effects are present whether they are balanced or extreme. Numbers listed here are the average Brier scores for drift selections.

D	T	B/E	AIC	WAIC	DIC	DIC_p	BIC	BF-BS	BF-TI	$ROPE_a$
L	L	B	1	1	1	1	1	1	1	1
L	L	E	1	1	1	1	1	1	1	1
L	M	B	1	1	1	1	1	1	1	1
L	M	E	1	1	1	1	1	1	1	1
L	S	B	1	1	1	1	1	1	1	1
L	S	E	1	1	1	1	1	1	1	1
M	L	B	0.99	0.99	0.99	0.91	0.9	0.95	0.93	0.97
M	L	E	0.99	1	1	0.93	0.88	0.85	0.87	0.98
M	M	B	1	1	1	0.98	0.95	0.95	0.94	0.99
M	M	E	0.99	1	1	0.96	0.9	0.96	0.94	0.99
M	S	B	1	1	1	1	0.99	0.99	0.99	0.99
M	S	E	0.98	0.98	0.99	0.92	0.89	0.91	0.89	0.96
S	L	B	0.2	0.37	0.37	-0.26	-0.42	-0.24	-0.29	-0.12
S	L	E	0.21	0.31	0.34	-0.32	-0.45	-0.37	-0.35	-0.17
S	M	B	0.38	0.49	0.52	-0.19	-0.37	-0.26	-0.26	-0.08
S	M	E	0.4	0.52	0.55	-0.19	-0.32	-0.24	-0.22	-0.1
S	S	B	0.15	0.37	0.41	-0.38	-0.6	-0.58	-0.55	-0.15
S	S	E	0.28	0.35	0.39	-0.17	-0.21	-0.09	-0.15	-0.23
L	N		1	1	1	1	1	1	1	1
M	N		1	1	1	1	1	1	1	0.98
S	N		0.57	0.7	0.72	0.07	-0.06	-0.02	-0.03	-0.22
N	L		0.45	0.19	0.12	0.88	0.97	0.78	0.84	0.83
N	M		0.39	0.06	-0.01	0.85	0.96	0.83	0.87	0.8
N	S		0.08	-0.07	-0.09	0.54	0.66	0.52	0.49	0.79
N	N		0.44	0.18	0.13	0.9	0.97	0.96	0.91	0.86

Table A5: Corresponds to Figure 5. The first three columns refer to the drift effect (null, small, moderate, large), threshold effect (null, small moderate, large), and when both effects are present whether they are balanced or extreme. Numbers listed here are the number of correct threshold identifications out of a possible 100.

D	T	B/E	AIC	WAIC	DIC	DIC_p	BIC	BF-BS	BF-TI	$ROPE_a$	DEV
L	L	B	100	100	100	100	100	100	100	100	100
L	L	E	100	100	100	100	100	100	100	100	100
L	M	B	100	100	100	100	100	100	99	100	100
L	M	E	99	99	99	99	98	98	97	99	99
L	S	B	78	83	81	48	35	40	41	61	57
L	S	E	79	83	84	50	34	24	25	60	59
M	L	B	100	100	100	100	100	100	100	100	100
M	L	E	100	100	100	100	100	100	100	100	100
M	M	B	100	100	100	100	99	99	99	100	100
M	M	E	100	100	100	99	97	97	98	98	100
M	S	B	73	80	81	50	39	35	43	57	54
M	S	E	77	78	80	49	33	30	29	48	55
S	L	B	100	100	100	100	100	100	100	100	100
S	L	E	100	100	100	100	100	100	100	100	100
S	M	B	100	100	100	100	100	99	99	100	100
S	M	E	100	100	100	100	100	99	99	100	100
S	S	B	66	74	76	27	16	14	11	51	62
S	S	E	82	85	82	64	59	52	54	53	67
L	N		85	74	76	97	99	99	99	95	94
M	N		78	70	72	96	99	99	98	91	95
S	N		75	72	71	89	92	96	93	95	86
N	L		100	100	100	100	100	100	100	100	100
N	M		100	100	100	100	100	99	100	100	100
N	S		84	84	83	73	66	54	54	50	81
N	N		87	80	79	98	99	100	100	98	91

Table A6: Corresponds to Figure 6. The first three columns refer to the drift effect (null, small, moderate, large), threshold effect (null, small moderate, large), and when both effects are present whether they are balanced or extreme. Numbers listed here are the average Brier scores for threshold selections.

D	T	B/E	AIC	WAIC	DIC	DIC_p	BIC	BF-BS	BF-TI	$ROPE_a$
L	L	B	1	1	1	1	1	1	1	1
L	L	E	1	1	1	1	1	1	1	1
L	M	B	1	1	1	1	1	0.99	0.97	1
L	M	E	0.98	0.98	0.98	0.96	0.94	0.93	0.9	0.98
L	S	B	0.48	0.58	0.57	-0.09	-0.29	-0.28	-0.26	-0.01
L	S	E	0.48	0.57	0.58	-0.09	-0.3	-0.4	-0.4	-0.03
M	L	B	1	1	1	1	1	1	1	1
M	L	E	1	1	1	1	1	1	1	1
M	M	B	1	1	1	1	0.98	0.97	0.96	1
M	M	E	0.99	1	1	0.97	0.93	0.92	0.93	0.97
M	S	B	0.43	0.54	0.56	-0.09	-0.28	-0.29	-0.25	-0.06
M	S	E	0.44	0.51	0.5	-0.12	-0.31	-0.38	-0.4	-0.13
S	L	B	1	1	1	1	1	1	1	1
S	L	E	1	1	1	1	1	1	1	1
S	M	B	1	1	1	1	0.99	0.98	0.98	1
S	M	E	1	1	1	1	1	0.98	0.97	0.99
S	S	B	0.27	0.42	0.43	-0.31	-0.55	-0.62	-0.62	-0.11
S	S	E	0.61	0.64	0.63	0.22	-0.01	-0.11	-0.09	-0.08
L	N		0.37	0.17	0.18	0.84	0.95	0.96	0.91	0.77
M	N		0.32	0.12	0.12	0.8	0.94	0.95	0.88	0.73
S	N		0.14	-0.01	0	0.6	0.76	0.87	0.77	0.81
N	L		1	1	1	1	1	1	1	1
N	M		1	1	1	1	1	0.99	1	1
N	S		0.65	0.63	0.63	0.28	0	-0.11	-0.12	-0.14
N	N		0.41	0.27	0.27	0.88	0.96	0.99	0.98	0.88

Table A7: Corresponds to Figure 7. The first two columns refer to the two methods being assessed for consistency in that row.

Method	Method	M/L-M/L	M/L-S_{bal}	M/L-S_{ext}	S-S_{bal}	S-S_{ext}	M/L-N	S-N	N-N
AIC	WAIC	1	0.9	0.9	0.82	0.85	0.84	0.77	0.73
AIC	DIC	1	0.91	0.9	0.79	0.86	0.84	0.74	0.73
AIC	DIC-p	0.99	0.73	0.67	0.52	0.63	0.86	0.72	0.8
AIC	BIC	0.98	0.6	0.57	0.4	0.6	0.84	0.68	0.79
AIC	BF-BS	0.98	0.64	0.54	0.41	0.55	0.82	0.61	0.78
AIC	BF-TI	0.98	0.64	0.56	0.4	0.57	0.85	0.61	0.78
AIC	ROPE-a	1	0.84	0.79	0.7	0.56	0.9	0.46	0.78
AIC	DEV	1	0.8	0.79	0.8	0.7	0.88	0.83	0.85
WAIC	DIC	1	0.97	0.98	0.97	0.92	0.96	0.96	0.91
WAIC	DIC-p	0.99	0.66	0.63	0.41	0.55	0.73	0.62	0.58
WAIC	BIC	0.98	0.54	0.52	0.3	0.51	0.71	0.55	0.57
WAIC	BF-BS	0.98	0.56	0.49	0.32	0.47	0.68	0.52	0.58
WAIC	BF-TI	0.98	0.57	0.52	0.3	0.49	0.72	0.51	0.57
WAIC	ROPE-a	1	0.78	0.74	0.64	0.5	0.77	0.39	0.58
WAIC	DEV	1	0.73	0.74	0.73	0.59	0.75	0.68	0.62
DIC	DIC-p	0.99	0.66	0.62	0.4	0.55	0.73	0.6	0.57
DIC	BIC	0.98	0.54	0.52	0.29	0.5	0.71	0.54	0.56
DIC	BF-BS	0.98	0.56	0.48	0.31	0.47	0.68	0.5	0.57
DIC	BF-TI	0.98	0.57	0.51	0.29	0.48	0.72	0.5	0.57
DIC	ROPE-a	1	0.78	0.73	0.61	0.49	0.77	0.38	0.57
DIC	DEV	1	0.73	0.73	0.72	0.6	0.75	0.66	0.61
DIC-p	BIC	0.98	0.86	0.89	0.85	0.93	0.98	0.88	0.99
DIC-p	BF-BS	0.99	0.86	0.84	0.85	0.88	0.95	0.84	0.98
DIC-p	BF-TI	0.98	0.83	0.77	0.82	0.83	0.96	0.8	0.96
DIC-p	ROPE-a	0.99	0.84	0.84	0.6	0.79	0.96	0.62	0.93
DIC-p	DEV	0.99	0.9	0.86	0.6	0.92	0.98	0.85	0.92
BIC	BF-BS	0.99	0.92	0.92	0.96	0.93	0.97	0.92	0.99
BIC	BF-TI	0.99	0.86	0.84	0.91	0.88	0.98	0.84	0.97
BIC	ROPE-a	0.98	0.75	0.78	0.46	0.78	0.94	0.69	0.94
BIC	DEV	0.98	0.8	0.78	0.46	0.9	0.96	0.82	0.91
BF-BS	BF-TI	0.99	0.87	0.85	0.95	0.9	0.95	0.86	0.98
BF-BS	ROPE-a	0.99	0.77	0.75	0.46	0.78	0.91	0.69	0.94
BF-BS	DEV	0.98	0.82	0.75	0.46	0.85	0.93	0.74	0.9
BS-TI	ROPE-a	0.98	0.76	0.74	0.45	0.79	0.93	0.68	0.92
BS-TI	DEV	0.98	0.8	0.73	0.44	0.85	0.94	0.73	0.9
ROPE-a	DEV	1	0.9	0.9	0.75	0.81	0.96	0.6	0.89

Table A8: Corresponds to Figure 8. The first two columns refer to the data type and prior type. Prop columns are the number of correct identifications out of a possible 100, and Brier columns are the average Brier scores.

Data	Prior	Prop Correct	Prop Drift	Prop Thres	Brier Correct	Brier Drift	Brier Thres
Bal	UP	1	1	1	-0.92	-0.93	-0.93
Bal	WIP	12	14	12	-0.55	-0.56	-0.65
Bal	MIP	35	48	40	0.05	0.09	-0.11
Bal	HIP	24	49	55	0.02	0	0.06
Ext	UP	0	51	47	-0.79	-0.16	-0.24
Ext	WIP	1	52	48	-0.54	-0.06	-0.13
Ext	MIP	71	75	96	0.49	0.46	0.85
Ext	HIP	92	92	100	0.48	0.27	0.96
Drift	UP	31	31	98	-0.22	-0.36	0.95
Drift	WIP	66	66	98	0.38	0.03	0.9
Drift	MIP	82	87	88	0.52	0.63	0.38
Drift	HIP	10	71	15	-0.09	0.11	-0.17
Thres	UP	29	89	30	-0.29	0.77	-0.45
Thres	WIP	49	80	51	0.05	0.45	-0.16
Thres	MIP	63	67	77	0.2	-0.06	0.53
Thres	HIP	44	44	99	0.19	-0.02	0.63
None	UP	100	100	100	1	1	1
None	WIP	98	98	100	0.94	0.94	0.99
None	MIP	85	90	94	0.42	0.33	0.72
None	HIP	29	54	61	0	0	0.01

Table A9: Corresponds to Figure 9. The first two columns refer to the data type and number of trials used per condition. Numbers listed here are the number of correct identifications out of a possible 100, with C columns being model identifications, D being drift identifications, and T being threshold identifications.

Data	Trials	C DIC	C AIC	C BIC	C BF	D DIC	D AIC	D BIC	D BF	T DIC	T AIC	T BIC	T BF
Bal	30	16	15	2	1	33	25	9	13	30	27	8	3
Bal	100	38	29	4	5	55	40	10	13	54	45	7	5
Bal	300	67	51	12	14	74	58	13	15	76	66	16	14
Bal	900	99	94	48	44	100	94	48	45	99	98	53	47
Ext	30	7	3	0	2	39	27	17	29	38	38	25	14
Ext	100	22	12	0	1	61	46	34	47	61	63	54	39
Ext	300	51	42	2	1	69	60	43	48	82	82	59	52
Ext	900	96	96	59	56	99	99	76	79	97	97	83	77
Drift	30	30	24	15	16	45	31	17	18	68	79	88	96
Drift	100	32	32	17	19	48	38	17	19	64	73	88	92
Drift	300	68	69	60	63	92	79	60	63	71	75	92	96
Drift	900	72	88	95	98	98	97	95	98	72	88	95	98
Thres	30	26	30	14	8	68	81	95	90	39	37	16	10
Thres	100	42	44	26	19	65	75	90	90	61	51	28	21
Thres	300	55	76	65	53	57	78	90	83	83	84	66	54
Thres	900	51	85	99	95	51	85	99	95	98	100	99	96
None	30	63	70	92	91	73	80	98	95	82	85	94	96
None	100	59	77	98	96	69	84	98	96	81	91	100	100
None	300	56	78	99	99	69	89	100	99	79	87	99	100
None	900	33	73	99	99	43	84	100	100	69	84	99	99

Table A10: Corresponds to Figure 10. The first two columns refer to the data type and number of trials used per condition. Numbers listed here are the average Brier scores, with C columns being model Brier scores, D being drift Brier scores, and T being threshold Brier scores.

Data	Trials	C DIC	C AIC	C BIC	C BF	D DIC	D AIC	D BIC	D BF	T DIC	T AIC	T BIC	T BF
Bal	30	-0.13	-0.16	-0.51	-0.56	-0.1	-0.16	-0.51	-0.48	-0.14	-0.17	-0.52	-0.63
Bal	100	0.06	-0.02	-0.56	-0.62	0.03	-0.07	-0.59	-0.56	0.02	-0.03	-0.56	-0.69
Bal	300	0.46	0.3	-0.53	-0.55	0.41	0.15	-0.6	-0.58	0.43	0.27	-0.55	-0.62
Bal	900	0.93	0.88	-0.03	-0.01	0.94	0.85	-0.22	-0.18	0.93	0.91	-0.09	-0.14
Ext	30	-0.12	-0.2	-0.5	-0.5	-0.03	-0.12	-0.4	-0.31	-0.05	-0.09	-0.37	-0.49
Ext	100	-0.02	-0.1	-0.53	-0.48	0.3	0.09	-0.23	-0.1	0.33	0.32	-0.1	-0.24
Ext	300	0.28	0.2	-0.58	-0.54	0.39	0.28	-0.21	-0.09	0.63	0.61	-0.01	-0.11
Ext	900	0.91	0.91	0.24	0.24	0.94	0.93	0.35	0.46	0.93	0.94	0.53	0.42
Drift	30	-0.02	-0.06	-0.28	-0.28	-0.02	-0.13	-0.44	-0.42	0.07	0.17	0.66	0.79
Drift	100	0.04	0.03	-0.31	-0.3	0.04	-0.06	-0.48	-0.44	0	0.15	0.68	0.8
Drift	300	0.34	0.39	0.27	0.33	0.72	0.57	-0.06	-0.02	0	0.14	0.76	0.87
Drift	900	0.5	0.62	0.91	0.95	0.97	0.96	0.87	0.93	0.2	0.37	0.87	0.93
Thres	30	-0.04	-0.02	-0.24	-0.42	0.09	0.28	0.73	0.62	-0.06	-0.1	-0.41	-0.57
Thres	100	0.01	0.09	-0.19	-0.32	-0.05	0.1	0.7	0.61	0.13	0.02	-0.36	-0.48
Thres	300	0.21	0.4	0.35	0.14	-0.09	0.08	0.66	0.52	0.63	0.65	0	-0.11
Thres	900	0.23	0.54	0.96	0.9	-0.06	0.29	0.93	0.85	0.97	0.99	0.96	0.91
None	30	0.18	0.29	0.72	0.76	0.15	0.38	0.81	0.77	0.33	0.39	0.78	0.87
None	100	0.19	0.32	0.87	0.85	0.13	0.36	0.88	0.82	0.36	0.48	0.95	0.97
None	300	0.13	0.33	0.95	0.96	0.13	0.44	0.97	0.96	0.27	0.41	0.96	0.99
None	900	0.03	0.31	0.97	0.98	-0.03	0.36	0.99	0.99	0.19	0.38	0.97	0.99

Table A11: Corresponds to Figure 11. The first three columns refer to the assessment method (proportion or Brier), what the assessment was for (correct model, drift, or threshold), and the data type. Numbers listed here are either the number of correct identifications out of a possible 20, or the average Brier scores.

Assess	Type	Data	AIC	WAIC	DIC	DIC_p	BIC	BF-BS	BF-TI	$ROPE_a$	DEV
Prop	C	None	19	16	16	19	20	18	20	19	19
Prop	C	Drift	8	7	7	6	6	7	9	10	7
Prop	C	Thres	12	7	7	14	16	13	13	11	13
Prop	C	Bal	19	19	20	19	19	20	20	20	19
Prop	C	Ext	12	12	12	7	8	8	6	11	9
Prop	D	None	19	18	19	20	20	18	20	19	19
Prop	D	Drift	18	18	18	13	11	12	14	19	17
Prop	D	Thres	12	7	7	14	16	13	13	11	13
Prop	D	Bal	19	19	20	19	19	20	20	20	19
Prop	D	Ext	13	13	13	9	10	10	8	14	11
Prop	T	None	19	17	16	19	20	20	20	19	19
Prop	T	Drift	10	8	8	13	15	15	15	11	10
Prop	T	Thres	20	20	20	20	20	20	20	20	20
Prop	T	Bal	20	20	20	20	20	20	20	20	20
Prop	T	Ext	19	19	19	18	18	18	18	17	18
Brier	C	None	0.45	0.33	0.27	0.89	0.97	0.88	0.97	0.66	
Brier	C	Drift	-0.22	-0.21	-0.25	-0.22	-0.23	-0.18	-0.09	-0.09	
Brier	C	Thres	0.04	-0.14	-0.13	0.39	0.54	0.25	0.3	0.01	
Brier	C	Bal	0.94	0.92	0.99	0.93	0.88	1	1	1	
Brier	C	Ext	0.47	0.36	0.5	-0.17	-0.17	-0.12	-0.16	0.28	
Brier	D	None	0.52	0.5	0.46	0.97	0.98	0.85	0.98	0.65	
Brier	D	Drift	0.72	0.79	0.78	0.05	-0.04	-0.01	0.17	0.73	
Brier	D	Thres	-0.15	-0.28	-0.27	0.08	0.3	-0.04	-0.02	-0.16	
Brier	D	Bal	0.91	0.88	0.99	0.9	0.82	1	1	1	
Brier	D	Ext	0.32	0.15	0.34	-0.22	-0.21	-0.16	-0.19	0.23	
Brier	T	None	0.53	0.36	0.21	0.87	0.98	0.99	0.99	0.7	
Brier	T	Drift	-0.28	-0.31	-0.35	-0.08	0.01	0.09	0.1	-0.17	
Brier	T	Thres	1	1	1	1	1	1	1	1	
Brier	T	Bal	1	1	1	1	1	1	1	1	
Brier	T	Ext	0.88	0.89	0.91	0.75	0.7	0.67	0.68	0.67	

Table A12: Corresponds to Figure 12. The first two columns refer to the two methods being assessed for consistency in that row.

Method	Method	None	Drift	Thres	Bal	Ext
AIC	WAIC	0.85	0.85	0.75	1	0.9
AIC	DIC	0.85	0.85	0.75	0.95	0.9
AIC	DIC-p	0.9	0.7	0.9	1	0.75
AIC	BIC	0.95	0.55	0.8	1	0.8
AIC	BF-BS	0.85	0.55	0.85	0.95	0.8
AIC	BF-TI	0.95	0.65	0.95	0.95	0.7
AIC	ROPE-a	1	0.85	0.95	0.95	0.85
AIC	DEV	1	0.85	0.95	1	0.85
WAIC	DIC	0.9	0.9	1	0.95	0.9
WAIC	DIC-p	0.85	0.6	0.65	1	0.75
WAIC	BIC	0.8	0.45	0.55	1	0.8
WAIC	BF-BS	0.75	0.45	0.6	0.95	0.8
WAIC	BF-TI	0.8	0.5	0.7	0.95	0.7
WAIC	ROPE-a	0.85	0.7	0.8	0.95	0.75
WAIC	DEV	0.85	0.7	0.7	1	0.85
DIC	DIC-p	0.85	0.65	0.65	0.95	0.75
DIC	BIC	0.8	0.5	0.55	0.95	0.8
DIC	BF-BS	0.75	0.5	0.6	1	0.8
DIC	BF-TI	0.8	0.55	0.7	1	0.7
DIC	ROPE-a	0.85	0.7	0.8	1	0.8
DIC	DEV	0.85	0.8	0.7	0.95	0.75
DIC-p	BIC	0.95	0.8	0.9	1	0.95
DIC-p	BF-BS	0.9	0.85	0.95	0.95	0.95
DIC-p	BF-TI	0.95	0.85	0.95	0.95	0.95
DIC-p	ROPE-a	0.9	0.7	0.85	0.95	0.75
DIC-p	DEV	0.9	0.8	0.95	1	0.9
BIC	BF-BS	0.9	0.95	0.85	0.95	1
BIC	BF-TI	1	0.85	0.85	0.95	0.9
BIC	ROPE-a	0.95	0.5	0.75	0.95	0.8
BIC	DEV	0.95	0.6	0.85	1	0.95
BF-BS	BF-TI	0.9	0.9	0.9	1	0.9
BF-BS	ROPE-a	0.85	0.55	0.8	1	0.8
BF-BS	DEV	0.85	0.65	0.9	0.95	0.95
BS-TI	ROPE-a	0.95	0.65	0.9	1	0.7
BS-TI	DEV	0.95	0.75	1	0.95	0.85
ROPE-a	DEV	1	0.8	0.9	0.95	0.8
